# White Participants’ Perceptions of Implicit Bias Interventions in U.S. Courts

**DOI:** 10.3390/bs15091269

**Published:** 2025-09-17

**Authors:** Megan L. Lawrence, Kristen L. Gittings, Sara N. Thomas, Rose E. Eerdmans, Valerie P. Hans, John E. Campbell, Jessica M. Salerno

**Affiliations:** 1 Department of Psychology, Cornell University, Ithaca, NY 14850, USA; kg595@cornell.edu (K.L.G.); ree49@cornell.edu (R.E.E.); jessica.salerno@cornell.edu (J.M.S.); 2 School of Interdisciplinary Forensics, Arizona State University, Glendale, AZ 85306, USA; snthoma9@asu.edu; 3 Cornell Law School, Cornell University, Ithaca, NY 14850, USA; valerie.hans@cornell.edu; 4 Justice Through Empirical Data Institute, Campbell Law LLC, St. Louis, MO 63105, USA; john@campbelllawllc.com

**Keywords:** implicit bias, bias interventions, juror perceptions, judicial instructions

## Abstract

**Objective:** U.S. courts have implemented interventions educating jurors about implicit bias, although evidence for their effectiveness remains limited. We explored public perceptions of these interventions that might influence their ability to improve trial fairness and identified psychological factors predicting such perceptions. **Hypotheses:** We hypothesized that certain psychological factors (i.e., political conservatism, psychological reactance, skepticism toward social scientists, implicit and explicit racial bias, advantaged-group identity management strategies) would predict support for implicit bias interventions in courts. **Method:** White participants (*N* = 1016)—some of whom watched an implicit bias intervention in one of two formats (educational video, judicial instructions)—provided their perceptions of implicit bias interventions, evaluated the intervention they watched (if applicable), and completed individual difference measures. **Results:** Overall, participants supported implicit bias interventions in both formats. However, political conservatism and other hypothesized individual difference measures were associated with less favorable perceptions. We further explored participants’ perspectives via a thematic content analysis of open-ended impressions of the interventions. **Conclusions:** Courts are adopting implicit bias interventions despite mixed research regarding their effectiveness and a limited understanding of how they are perceived. Our findings suggest that White participants generally favor these interventions and offer insight into the nuances of their perceptions.

## 1. Perceptions of Implicit Bias Interventions in U.S. Courts

The right to a fair and impartial trial is a core tenet of the United States legal system, and jurors take their job to return an impartial verdict very seriously (e.g., Diamond & Hans, 2023; Hans et al., 2024). Yet, the knowledge, beliefs, and experiences that jurors bring into the courtroom can potentially shape how they perceive evidence and bias their judgments. The role of bias in juror decision-making is well documented (e.g., Devine & Caughlin, 2014; Mitchell et al., 2005) and aligns with decades of social psychological research demonstrating that people make biased decisions even when they are motivated to avoid doing so (Paluck & Green, 2009).

One explanation for this phenomenon is that jurors are influenced by their *implicit* biases, which can occur without their awareness (Greenwald & Banaji, 1995). In some instances, implicit biases can be even more predictive of biased behavior than self-reported, explicit attitudes (for a meta-analytic review, see Greenwald et al., 2009). Scientists and legal experts have long expressed concern about how implicit bias might impact jurors’ decisions in both criminal and civil courts (Kang et al., 2012).

To address this threat to fairness, many U.S. courts at the federal, state, and district levels have poured extensive time and resources into developing and implementing interventions to educate jurors about implicit bias (e.g., Federal Judicial Center, n.d.; New Jersey Courts, 2022; Western District of Washington, 2021). These educational interventions, often developed in partnership with research organizations (e.g., Perception Institute, n.d.) or implicit bias experts (e.g., Coward, 2023), explain this topic more deeply than that found in the brief statements about bias included in most standard judicial instructions. Specifically, they explain the concept of implicit bias using real-world examples, emphasize the importance of making decisions without implicit bias, and offer jurors some general strategies to monitor and control the influence of implicit bias on their decisions.

Many courts across the U.S. have adopted some sort of implicit bias intervention for jurors (National Center for State Courts, n.d.). These interventions are most often delivered through a short, court-produced educational video or instructions from the judge (Kirshenbaum & Miller, 2021). Depending on the jurisdiction, they might be delivered during the jury selection process, in preliminary instructions, in closing instructions before deliberation, or at multiple timepoints throughout the trial (Western District of Washington, 2021).

Because they are already frequently being used in courtrooms today, it is essential to investigate both (1) whether implicit bias interventions *actually* achieve their intended goal of improving trial fairness by reducing the impact of bias on jurors’ case decisions and (2) how jurors react psychologically to these interventions. We investigated the first question in an experimental context, which we fully report in another article (Lawrence et al., 2025) and describe in detail in the next section. We found some promising evidence that judicial instructions improved civil trial outcomes for Black plaintiffs, while educational videos did not—and neither form of implicit bias intervention mitigated the relationship between jurors’ racial bias and their case decisions about Black plaintiffs. This aligns with the broader social psychological literature, which indicates that interventions targeting implicit bias are generally ineffective at reducing bias (e.g., Forscher et al., 2019; Lai et al., 2016; Lewis, 2023).

Here, we explore a second question: How do jurors react psychologically to these interventions? This question is important for several reasons—particularly given that the general concept of implicit bias (Bagenstos, 2018; Reid et al., 2025) or feedback about implicit bias (Lofaro et al., 2024) can elicit resistance. First, we can investigate whether people react negatively to the interventions in ways that might limit their effectiveness, potentially providing courts with insight on how to refine them. Second, we can investigate whether people overestimate their effectiveness. If jurors and courts believe that these brief interventions are sufficient to address bias, it may divert attention away from pursuing more robust strategies. We preregistered a plan to collect and analyze a number of exploratory judgments and psychological factors that were not reported in the first article (Lawrence et al., 2025) but provide insight into this novel second question. Specifically, we assessed laypeople’s general perceptions of implicit bias interventions in courts and, when applicable, their evaluations of the specific intervention they watched (i.e., an educational video or judicial instructions). Further, we assessed individual differences that may be associated with more or less support for bias education in court settings to identify who might react negatively to the interventions.

### 1.1. The Impact of Implicit Bias Interventions on Juror Decision-Making

While the current article focuses on laypeople’s perceptions of implicit bias interventions, it is important to consider these perceptions in the broader context of whether these interventions achieve their intended goal of promoting trial fairness. A substantial body of research outside of the courtroom context has demonstrated that, in some cases, implicit biases may be reduced through an intervention. However, these effects generally stem from curated laboratory scenarios that are not applicable to real-world courtrooms (e.g., exposing people to counter-stereotypical exemplars; Lai et al., 2014, 2016), are not long-lasting (Lai et al., 2016), and do not necessarily lead to a reduction in biased behavior (Forscher et al., 2019). Further, interventions that are most similar to those used in courts—such as priming feelings of egalitarianism (e.g., similar to instructing jurors to be fair and impartial)—have been found to be ineffective, even in the short term (Lai et al., 2014, 2016). Most studies that tested educational interventions about implicit bias examined their influence on self-reported bias awareness, measured bias scores, or *intentions* of behavior change (Atewologun et al., 2018). Few have measured behavior change directly—and those that do rarely show evidence that the intervention was effective (for an exception, see Forscher et al., 2017).

A few mock juror experiments have tested implicit bias interventions in courtroom contexts. One study found that watching an educational video about implicit bias did not reduce the biasing effects of being exposed to pre-trial publicity on mock jurors’ verdicts and other related judgments (Jones et al., 2022). Most subsequent mock juror experiments tested the effect of implicit bias interventions on judgments about Black versus White criminal defendants (e.g., Lynch et al., 2022; Ruva et al., 2024). However, these researchers did not find evidence of anti-Black racial bias in case judgments to begin with, making it impossible to evaluate whether the interventions had the potential to mitigate that bias. These studies were conducted in a criminal context where racial bias is very salient (Gonzalez-Barrera et al., 2024), which may have exacerbated social desirability concerns and reduced mock jurors’ willingness to return verdicts against a Black defendant (see Salerno et al., 2023).

In an attempt to avoid such heightened social desirability concerns, we conducted a similar experiment in a *civil* trial context, fully reported in a prior article (Lawrence et al., 2025) and summarized here. We randomly assigned White participants to (a) watch an educational video about implicit bias, (b) watch a video of instructions about implicit bias delivered by a judge, or (c) not watch a video about implicit bias before judging a mock civil case with either a Black or White plaintiff. We recruited a White sample in an attempt to increase the likelihood that we would get racial bias effects that align with field data, which we explain in greater detail in Section 2. As we hypothesized, as White mock jurors’ explicit racial biases increased, they delivered less favorable verdicts for Black, but not White, plaintiffs. Unfortunately, neither implicit bias intervention mitigated this relationship. We found one promising effect of judicial instructions, specifically: White mock jurors were 1.65 times more likely to award a favorable verdict to a Black plaintiff when they received judicial instructions about implicit bias than when they received only standard judicial instructions—but the instructions had no effect on the judgments of a White plaintiff. Educational videos, however, did not lead to more favorable judgments for Black or White plaintiffs.

Given that the judicial instructions did not mitigate the link between mock jurors’ racial bias and verdicts for a Black plaintiff, it is difficult to know whether this finding reflects actual bias reduction or merely a social desirability bias. Regardless, this is promising and should be tested in future research. Notably, other areas of psycholegal research have found that pre-trial instructions have limited, if any, debiasing effects on jurors’ judgments. For example, contrary to judges’ instructions, jurors struggle not to draw any guilt inferences when a defendant exercises their right not to testify (e.g., Frank & Broschard, 2006). Additionally, decades of experimental data find that mock jurors’ case decisions are influenced by inadmissible evidence despite judges’ instructions to disregard it (meta-analyses: Nietzel et al., 1999; Steblay et al., 2006).

In summary, the broader literature offers little evidence that implicit bias interventions decrease the influence of bias on decision-making, which raises the question of *why* they may be ineffective. Our main experimental findings also left us with some unanswered questions, including why judicial instructions, but not educational videos, impacted mock jurors’ verdicts for Black plaintiffs (Lawrence et al., 2025). While the primary goal of our experiment was to test the interventions’ impact on case outcomes, we also included exploratory measures to address a related, but distinct, secondary question: How do mock jurors *perceive* implicit bias interventions? We reasoned that examining what participants were taking away from the interventions might (a) explain why one intervention, but not the other, made mock jurors’ verdicts more favorable to Black defendants and (b) identify psychological factors that help explain why some jurors might support these interventions more than others.

### 1.2. Perceptions of Implicit Bias Interventions

If academics or court administrators have misassumptions about jurors’ perceptions of implicit bias interventions, they may have unintended effects. For instance, consider alcohol-related interventions among college students. In a large-scale survey, over half of college students rated alcohol-related experiences that might be assumed to be negative—such as hangovers, blackouts, or social embarrassment—as neutral or even positive (Mallett et al., 2008). Interventions based on the assumption that college students perceive these experiences as negative might fail to resonate with college students and, as a result, have a limited effect on their behaviors.

The same principle applies in legal settings: If jurors perceive anti-bias interventions differently than the courts intend, this could limit their ability to address juror bias in case outcomes. Given that the broader literature finds limited evidence that educational interventions about implicit bias can mitigate biased decision-making, exploring participants’ perceptions of implicit bias interventions may begin to address some unanswered questions about why this might be the case. For instance, if laypeople found judicial instructions more relevant or important than educational videos, this may explain why we found that judicial instructions, but not educational videos, improved outcomes for Black plaintiffs. Jurors rely on judges to help them apply the law fairly (Diamond & Hans, 2023), and their messages might better resonate with jurors than those shared in a routine educational video shown in all cases.

In fact, people’s ability to correct for their own biases largely depends on their motivation to do so (Fazio & Towles-Schwen, 1999), and legal scholars have argued that educating jurors about the science of implicit bias may increase their motivation to monitor their own biases (Kang et al., 2012). For an intervention to be effective, it is important that people want to learn and apply its content—which is shaped by the extent to which they perceive the intervention as relevant, useful, and enjoyable to participate in (Gegenfurtner et al., 2009). Research in other industries (e.g., education: Lindvall-Östling, 2024; healthcare: Sherman et al., 2019) and among other law-related populations (i.e., judges: Giannetta et al., 2023; Kirshenbaum & Miller, 2021; police officers: Lai & Lisnek, 2023; Worden et al., 2020) suggests that people who are exposed to implicit bias interventions generally report positive reactions to them. While positive perceptions of implicit bias interventions do not guarantee that they will be effective, they may lead jurors to at least be open to putting effort into learning and applying their content.

Critically, as judges and legal scholars have noted, implicit bias interventions must avoid triggering defensiveness or feelings of insult among jurors (Kang et al., 2012; Kirshenbaum & Miller, 2021). However, being told that one is biased—a key feature of the interventions—has been found to trigger defensive reactions (Howell & Ratliff, 2017; Vitriol & Moskowitz, 2021). Even general discussions about bias and inequality can evoke feelings of defensiveness and threat among advantaged-group members (Knowles et al., 2014; Shuman et al., 2024). Unfortunately, anti-prejudice messaging can have counterintuitive effects, depending on how it is framed and received. One experiment, for example, found that anti-prejudice messaging that used controlling language (e.g., “In today’s society, you must control prejudice”) or emphasized social norms (e.g., “the better we are at reducing prejudice, the more likely we are to fit in”) ironically *increased* the expression of biased behaviors (Legault et al., 2011). The authors expressed concern that anti-bias messages may be “worse than doing nothing at all” if they are not framed properly, perhaps because such controlling language may “incit[e] hostility” or “a desire to rebel against prejudice reduction itself” (Legault et al., 2011, p. 1476). In a time when diversity, equity, and inclusion-related efforts have been attacked in the broader sociopolitical climate (Trump, 2025), it is reasonable to expect that implicit bias interventions could trigger defensive reactions from some individuals.

Notably, however, two features of implicit bias interventions may decrease the likelihood of defensive responding. First, the interventions normalize bias as a human factor to decrease moral blameworthiness. Second, they give jurors the impression that they are able to control their biases by providing general advice or strategies (e.g., Oregon Courts, 2020). These features have been associated with decreased defensiveness to bias feedback (Vitriol & Moskowitz, 2021). However, research on lay perceptions of implicit bias interventions has thus far been limited, and, to our knowledge, nonexistent in courtroom contexts.

### 1.3. Individual Differences in Perceptions of Implicit Bias Interventions

Some jurors might have more positive reactions to implicit bias interventions than others, as they bring their own experiences, beliefs, and attitudes into the courtroom. Individual differences in ideology and perspective often interact with situational factors in the courtroom to impact juror behavior (Rae & Greenwald, 2017), making it particularly important to examine their role in court contexts. Research that has explored how bias operates has extensively focused on the role of individual characteristics (e.g., right-wing authoritarianism, social dominance orientation; Duckitt & Sibley, 2007). Perhaps because they are relatively new, however, research about bias education programs has largely focused on general psychological processes that apply to everyone (e.g., counterstereotypic exemplars: Finnegan et al., 2015; metacognition about bias: Sabin et al., 2022), paying less attention to individual differences. We aimed to integrate these approaches by considering how individual differences shape jurors’ perspectives.

At a time when diversity- and equity-based initiatives are under attack (e.g., Trump, 2025), it is especially important to investigate how these interventions may be received across people who may differ in their ideology and perspectives. Ultimately, it is up to the individual to decide what to take away from an intervention, if anything, which highlights the need to explore individual differences more deeply (Ford et al., 2018; Mathieu & Martineau, 2014). Considering that negative perceptions of implicit bias interventions might limit their effectiveness, we aimed to advance the theoretical understanding of when bias education might be effective by identifying who reacts positively or negatively. We investigated whether a variety of theoretically relevant individual difference measures (i.e., political orientation, advantaged-group identity management, skepticism of social scientists who study race, psychological reactance, racial bias) predicted the degree to which participants support implicit bias interventions in court settings, which we describe next.

#### 1.3.1. Political Orientation

Differences in perspectives around equity-driven initiatives tend to fall along political lines. For example, Republicans tend to view diversity, equity, and inclusion (DEI) initiatives in the workplace more negatively than Democrats (Minkin, 2024). Republican and conservative groups have also led efforts to restrict DEI initiatives through legislation (e.g., Goldberg, 2024; Iyer & Boyette, 2023) and litigation (e.g., Palmer, 2024; Sheen, 2023). Relative to liberals, conservatives are more resistant to the general concept of implicit bias (Bagenstos, 2018) and are less likely to report that implicit bias against people of color is a major problem in the United States legal system (Reid et al., 2025). Thus, we hypothesized that greater political conservatism would be associated with less support for implicit bias interventions.

#### 1.3.2. Advantaged-Group Identity Management

Messages about group-based inequalities, which are included in educational videos about implicit bias, may cause feelings of status threat and defensiveness among members of historically advantaged groups (Knowles et al., 2014; Shuman et al., 2024). Advantaged-group members might employ the following to resolve these feelings of threat: (a) *defend*, or justify, the inequalities as natural or deserved; (b) *deny* that the inequalities actually exist; (c) *distance* themselves from the inequality or from their status as an advantaged-group member; or (d) *dismantle* the inequality by supporting policies and actions that reduce their in-group privilege. Out of these four approaches, implicit bias interventions most closely align with the *dismantle* strategy, as they aim to reduce group-based inequalities in case outcomes through bias education. However, individuals who prefer to *defend*, *deny*, or *distance* themselves from the inequality may reject implicit bias education, as the interventions are grounded in social science about bias and disparities that may conflict with their beliefs. Thus, we hypothesized that decreased endorsement of *dismantle* strategies, but *increased* endorsement of *defend*, *deny*, or *distance* strategies, would be associated with less support for implicit bias interventions.

#### 1.3.3. Skepticism of Social Scientists

Historically, Americans have had relatively high trust and confidence in scientists (National Science Board, 2024), with over 85% of surveyed U.S. adults reporting “some” or “a great deal” of confidence in the scientific community annually from 2002 to 2022 (Lupia et al., 2024). Yet, Americans—especially Republicans—have reported increasing distrust toward scientists following the COVID-19 pandemic (Tyson & Kennedy, 2024). In a recent survey, only 42% of respondents across the political spectrum expressed some level of agreement with the statement that scientists can “overcome their human and political biases” (Lupia et al., 2024). This has occurred alongside political attacks on scientists and scientific research across disciplines (Dajches, 2025). Because implicit bias interventions are based on social science research about bias (FitzGerald et al., 2019), individuals who are distrustful of social scientists may have defensive or negative reactions to them. We hypothesized that greater skepticism of social scientists who study race issues, in particular, would be associated with less support for implicit bias interventions.

#### 1.3.4. Psychological Reactance

Psychological reactance is a trait-level characteristic that can exacerbate feelings of defensiveness when an individual feels threatened (Dillard & Shen, 2005). Some evidence suggests that increased psychological reactance may decrease people’s willingness to engage in anti-bias behaviors, particularly after receiving feedback about their own bias (Lofaro et al., 2024). Thus, we hypothesized that greater psychological reactance would be associated with less support for implicit bias interventions.

#### 1.3.5. Racial Bias

We also explored whether participants’ racial biases—measured both implicitly and explicitly—were associated with support for implicit bias interventions. Although individuals who are relatively more biased might perceive the interventions as necessary, they may also respond more defensively. We did not preregister specific hypotheses about these two exploratory measures.

### 1.4. Research Overview

The extent to which an intervention is capable of having its intended effect, in part, depends on people’s perceptions of that intervention (Ford et al., 2018). It is especially timely to investigate laypeople’s perceptions of implicit bias interventions because they are being used in courtrooms across the U.S. (National Center for State Courts, n.d.), despite limited evidence of their effectiveness. In this study, we explored White participants’ perceptions of implicit bias interventions in two ways. First, using Likert-style questions, we investigated the extent to which participants support the use of implicit bias interventions in courts, tested which individual differences predict that support, and compared perceptions of educational videos and judicial instructions about implicit bias. Second, we further probed participants’ perceptions by asking them two broad open-ended questions: one about the interventions generally, and another about the specific intervention they watched, if applicable. Using a thematic content analysis, we explored themes in participants’ evaluations of the interventions’ content, their concerns about the interventions, and their beliefs about why courts implemented them.

#### Transparency and Open Science Practices

This study was conducted under an exemption granted by the Institutional Review Board at Arizona State University (Protocol No. 0001858). Our hypotheses and measures for this study were preregistered on the Open Science Framework (OSF), available at this link (https://osf.io/gc6x2).

As noted, the current article addresses a secondary question via measures collected within a broader experiment. Specifically, we randomly assigned White participants to (a) watch an educational video about implicit bias, watch a video of anti-bias instructions delivered by a judge from the bench, or not watch an implicit bias intervention, and then (b) judge a mock civil case with either a Black or White plaintiff. In another article, we report the impact of the interventions on mock jurors’ case decisions (Lawrence et al., 2025). Here, we focus on perceptions of the interventions. Importantly, each article has different theoretical aims, as indicated by separate, preregistered hypotheses (see OSF) and entirely non-overlapping outcome measures.

We were mindful of the potential impact of the broader experiment’s manipulations on the key measures relevant to this article. To address this directly, we tested whether participants’ responses to 14 total measures (i.e., quantitative evaluations of the interventions and all individual difference predictors) differed based on participants’ experimental conditions. The plaintiff race manipulation did not significantly affect any of the measures (see Supplemental Materials, Tables S3 and S4). 

The implicit bias intervention condition (educational video, judicial instructions, none) affected only two measures (see Supplemental Materials, Tables S5 and S6), both of which are theoretically expected. First, participants who viewed either intervention (judicial instructions, educational video) expressed greater support for their use in courts than participants who did not receive an intervention. Participants in the no-intervention condition may have had relatively less favorable perceptions because they relied on their prior experiences or attitudes to form their judgments. It is possible that the training description led them to think of diversity, equity, and inclusion trainings, generally, which are often polarized (Minkin, 2024; Palmer, 2024; Sheen, 2023). Second, participants who watched the educational video reported less psychological reactance than those who did not watch the intervention. This scale partly captures perceptions of whether implicit bias interventions are useful for them. The educational videos emphasize that everyone is subject to implicit biases, which may have reduced resistance to bias education. The judicial instructions convey this more briefly, which might explain why this effect did not extend to this condition.

In summary, we found no evidence that the experimental manipulations influenced participants’ judgments in ways that would call our results into question. We fully report these analyses in the Supplemental Materials (Tables S3–S6).

## 2. Method

### 2.1. Participants

Our target sample size (*N* = 1059) was determined based on a power analysis for our primary research question that tested the impact of implicit bias interventions on case judgments (Lawrence et al., 2025; see OSF). We recruited 1332 White participants using Connect by CloudResearch, targeting an equal number of conservative and liberal participants to capture variation in ideological attitudes. We first excluded participants who completed the survey more than once (*n* = 26), did not complete the survey (*n* = 65), or identified as a race other than White (*n* = 14). Next, we excluded participants who showed signs of inattention by completing certain aspects of the survey unreasonably quickly or failing various attention and manipulation checks^1^ associated with the broader experiment (*n* = 211).

Notably, our sample consisted only of participants who self-identified as White. This decision was based on the primary goal of the broader experiment: to test the effect of implicit bias interventions on mock jurors’ decisions in cases with Black and White plaintiffs (Lawrence et al., 2025). Specifically, we aimed to experimentally demonstrate an anti-Black bias effect consistent with field data showing that Black plaintiffs receive less favorable trial outcomes than plaintiffs of other racial groups (e.g., Chin & Peterson, 1985; Girvan & Marek, 2016). Prior research has demonstrated that mock jurors often show in-group racial bias effects in experiments (meta-analysis: Mitchell et al., 2005). This led us to focus on White participants’ potential outgroup bias toward Black plaintiffs to increase the likelihood of being able to test whether the implicit bias interventions mitigated it. This sampling choice, however, prevented us from examining how people of color perceive or respond to implicit bias interventions—a point we address in Section 4.4 and view as a critical direction for future work.

Our final sample consisted of 1016 White participants, ranging from 18 to 82 years old (*M* = 41.95, *SD* = 13.35). The participants identified as women (58.5%), men (40.2%), or nonbinary/other (1.4%). On a 6-point scale, the participants self-identified as very liberal (19.5%), somewhat liberal (22.6%), slightly liberal (10.5%), slightly conservative (13.2%), somewhat conservative (22.3%), or very conservative (11.8%).

### 2.2. Materials

#### 2.2.1. Implicit Bias Interventions

In the broader experiment (Lawrence et al., 2025), some mock jurors were randomly assigned to watch an implicit bias intervention in the form of either an educational video (*n* = 319) or a video of instructions delivered by a judge (*n* = 330). We used stimulus sampling (Wells & Windschitl, 1999). Participants who were randomly assigned to the educational video condition watched one of four court-produced videos about implicit bias (Harris County District Clerk, 2019; New Jersey Courts, 2022; New York State Unified Court System, 2021; Western District of Washington, 2021). Participants who were randomly assigned to the judicial instructions condition watched a video of the same White female mock judge reading one of four sets of instructions about implicit bias from the bench in a mock courtroom (American Bar Association, 2017; *Minnesota v. Chauvin*, 2021; New Jersey Courts, n.d.; Western District of Washington, 2021). Content comparisons between these eight interventions are detailed in the Supplemental Materials (Tables S1 and S2).

#### 2.2.2. Measures

Example items from measures relevant to the current study are provided below, with all scale items available on OSF. With the exception of implicit and explicit racial bias measures, none of the following were reported in the prior article about mock jurors’ case decisions (Lawrence et al., 2025).

**Perceptions of Implicit Bias Interventions.**

***General Perceptions of Implicit Bias Interventions***. All participants—including those who did not view an implicit bias intervention video—were given a brief description of the general content of implicit bias interventions and were informed that implicit bias interventions are being used in U.S. courts today.

They then answered the following open-ended question: “What do you think motivated the courts to adopt these interventions? What are your opinions and thoughts about the use of implicit bias interventions in the courts?”^2^

Using 7-point scales (*strongly disagree* to *strongly agree*, with *neither agree nor disagree* as a midpoint), participants then indicated the extent to which they agreed with four items that reflect potential reasons someone might *support* implicit bias interventions (e.g., “Implicit bias interventions show people that the courts care about impartiality”) and four items reflecting reasons someone might *not support* implicit bias interventions (e.g., “Implicit bias interventions are a waste of the court’s time and our tax dollars”). All eight items are listed in Table A3.

***Overall Support for Implicit Bias Interventions.*** Participants were asked, “Do you support or oppose the use of implicit bias interventions in real trials?” and responded on a 6-point scale (*strongly oppose*, *oppose*, *somewhat oppose*, *somewhat support*, *support*, *strongly support*).

***Evaluation of a Specific Implicit Bias Intervention.*** Participants who were randomly assigned to watch an implicit bias intervention (*n* = 649) were given the opportunity to watch the video again and then responded to the following open-ended question: “In a few sentences, what was your impression of this video? What did you learn or take away from the video?”

Participants then answered six exploratory questions about the intervention they watched (i.e., educational video or judicial instructions)—specifically, the extent to which they agreed that the intervention was informative, engaging, helpful, confusing, misguided, and unscientific (e.g., *not at all helpful*, *slightly helpful*, *moderately helpful*, *very helpful*, *extremely helpful*). Because we used stimulus sampling, these responses captured evaluations of eight total interventions, rather than idiosyncratic features of a single intervention.

**Individual Difference Predictors.**

***Psychological Reactance.*** To assess participants’ psychological reactance to bias interventions, we slightly modified three existing items (Lofaro et al., 2024) and developed three novel, face-valid items to capture participants’ reactance to the interventions, including their willingness to consider whether the interventions could be useful for them. These six randomized items were assessed on a 6-point agreement scale (*strongly disagree* to *strongly agree*). Example items include, “I consider advice about how to not be biased from others to be an intrusion,” and “I feel open to thinking about how implicit bias might influence my behavior (R).” Select items were reverse scored, such that higher responses reflected greater reactance to implicit bias interventions. Participants, on average, somewhat disagreed with these items, demonstrating a low level of psychological reactance to implicit bias interventions (*M* = 2.32, *SD* = 0.94; α = 0.82).

***Advantaged-Group Identity Management Strategies.*** We targeted an all-White sample, which allowed us to capture the strategies participants use to manage their advantaged-group identity as White Americans in the context of White-Black relations in the U.S. (Shuman et al., 2024). On a 7-point scale, participants indicated the extent to which they agreed with a total of 15 randomized items (*strongly disagree* to *strongly agree*, with a midpoint of *neither agree nor disagree*), completing three items for each of the following five scales.

The *deny* scale captures the extent to which participants deny the existence of intergroup inequality (e.g., “The amount of inequality between Black and White Americans is actually quite small”). Participants, on average, responded near the midpoint on these items (*M* = 3.28, *SD* = 1.87; α = 0.94), indicating only a slight tendency toward acknowledging, rather than denying, intergroup inequality.

The *defend* scale captures the extent to which participants defend in-group privileges as natural and deserved (e.g., “The inequalities that exist between Black and White Americans are a justified outcome of the real differences between the groups”). Participants, on average, disagreed with these items (*M* = 2.27, *SD* = 1.37; α = 0.85), indicating that they generally do not believe that in-group privileges are natural or deserved.

The *distance* (*identity*) scale captures the extent to which participants distance themselves from their identity as an advantaged-group member (e.g., “It bothers me when people focus on my racial identity rather than on the things that define me as an individual”). Participants, on average, somewhat agreed with these items (*M* = 5.12, *SD* = 1.26; α = 0.71), indicating that participants generally distance themselves from their identity as a White American.

The *distance* (*inequality*) scale captures the extent to which participants distance themselves from the benefits or privileges afforded to advantaged-group members (e.g., “I have never personally benefited from the inequality that exists between Black and White Americans, although others might have”). Participants, on average, responded near the midpoint on these items (*M* = 3.79, *SD* = 1.51; α = 0.78), indicating only a slight tendency to distance themselves from the benefits or privileges afforded to White Americans.

The *dismantle* scale captures the extent to which participants support efforts to dismantle inequality through policies that reduce their in-group privilege (e.g., “To achieve equality for Black Americans, White Americans should be willing to give up some of the advantages they enjoy”). Participants, on average, responded near the midpoint on these items (*M* = 3.88, *SD* = 1.75; α = 0.90), indicating only a slight tendency to disagree with policies that reduce in-group privilege.

***Implicit Racial Bias.*** We captured participants’ implicit racial bias using the Race (Black-White) Implicit Association Test (IAT; Greenwald et al., 1998). We analyzed the speed and accuracy with which participants sorted Black and White faces with positive and negative words using iatgen software (v1.6.0; Carpenter et al., 2019). Participants, on average, showed moderate anti-Black implicit bias (*M* = 0.45, *SD* = 0.39; for IAT cutoffs, see Ratliff & Smith, 2021).

***Explicit Racial Bias.*** We captured explicit racial bias using the 6-item Bayesian Racism Scale (Uhlmann et al., 2010). On 7-point agreement scales (*strongly disagree* to *strongly agree*, with a midpoint of *neither agree nor disagree*), participants indicated the extent to which they believe that discriminatory behaviors based on racial stereotyping are appropriate in various situations (e.g., “If your personal safety is at stake, it’s sensible to avoid members of ethnic groups known to behave more aggressively”). Participants, on average, somewhat disagreed that stereotype-based discrimination is rational (*M* = 2.94, *SD* = 1.26; α = 0.82).

***Skepticism of Social Scientists.*** Modified from previous research (McCright et al., 2013), participants indicated the extent to which they trust or distrust social scientists who investigate racial bias and ethnic disparities. They responded to four randomized items that were assessed on a 5-point scale (*completely distrust*, *partially distrust*, *neither distrust nor trust*, *partially trust*, *completely trust*). An example item reads, “To what extent do you trust social scientists who investigate racial bias and ethnic disparities to create knowledge that is unbiased or accurate?” All items were reverse scored, such that higher values reflect greater skepticism. Participants, on average, reported high trust in social scientists (*M* = 1.40, *SD* = 1.07; α = 0.96).

#### 2.2.3. Additional Materials and Measures

Participants answered various attention check and manipulation check questions throughout the survey.^3^ Participants were also exposed to additional materials related to a mock civil case about a trip-and-fall incident in a store, featuring either a Black or White plaintiff, and completed measures that are not relevant to this manuscript, including case judgments (e.g., verdict, damage awards). We provide these materials on OSF (available here: https://osf.io/dfpj7/) but do not discuss these additional measures further.

### 2.3. Procedure

#### 2.3.1. Data Collection

Participants were randomly assigned to (a) watch an educational implicit bias video, watch a video of a judge reading implicit bias instructions, or not watch an implicit bias intervention, and then (b) read about a trip-and-fall civil case with either a Black or White plaintiff before returning their case judgments. All participants were then informed that U.S. courts are using implicit bias interventions in their procedures and received a short summary of their content. Participants then answered an open-ended question and a set of Likert-style questions about their general perceptions of implicit bias interventions. Next, participants in the intervention conditions (educational video, judicial instructions) were given the opportunity to watch their assigned intervention again before providing their open-ended impressions of the video and then rating the intervention on a variety of dimensions, such as whether the intervention was engaging, confusing, or helpful. Participants then completed measures that captured individual differences in the following order: psychological reactance, advantaged-group identity management strategies, implicit racial bias, explicit racial bias, and skepticism of social scientists studying race. Finally, participants provided basic demographic information (e.g., political orientation) and were debriefed.

#### 2.3.2. Codebook Development and Procedure

We conducted a thematic content analysis following our data collection (Naeem et al., 2023) to explore patterns in participants’ responses to the two open-ended questions. A thematic content analysis is a popular approach to identify themes in data that are theoretically relevant and occur with some level of frequency (Braun & Clarke, 2006). Specifically, we identified themes related to participants’ general impressions of implicit bias interventions and their evaluations of the specific intervention that they watched, if applicable (i.e., judicial instructions, educational video). Open-ended responses provide a more holistic view of participants’ perceptions. Specifically, they can capture participants’ spontaneous thoughts, reveal perspectives that may have otherwise been overlooked, and generate questions for future research (Power et al., 2018).

We used OpenAI’s (2024) ChatGPT-4o to identify initial patterns in responses to each question and refine these categories for systematic coding, as artificial intelligence is often used to streamline this process (Christou, 2024). OpenAI provided initial suggestions to ensure that the themes were well-defined and non-overlapping. We used these suggestions as a foundation to develop the codebook and made adjustments to capture additional themes that were of interest to our team. In other words, we started with a data-driven approach and supplemented this with theoretically-driven themes (Braun & Clarke, 2006). We detail this iterative process in the Supplemental Materials. Additionally, at the request of a reviewer, we explored the extent to which AI-generated themes overlapped between participants in the two plaintiff race conditions (Black, White). For both open-ended questions, there was strong conceptual overlap between these groups (see Supplemental Materials, Table S7). We proceeded with developing and reporting the themes generated on the full dataset.

Four independent coders—two for each question—recorded whether the themes were present in each participant’s responses. Coders extracted themes based only on what was written (i.e., a semantic approach), rather than interpreting underlying ideas or implications in the responses (i.e., a latent approach; Braun & Clarke, 2006). Across all responses, coders showed acceptable agreement across themes about general perceptions of implicit bias interventions in the first prompt (*M* = 96.1%; *median* = 98.7%; *range*: 75.5–99.8%) and about the specific intervention participants watched in the second prompt (*M* = 92.9%; *median* = 95.1%; *range*: 76.9–98.6%). We report percentage agreement for each theme in the Supplemental Materials (see Tables S8 and S9). Discrepancies were resolved via discussion, and we revised the codebooks as needed. We provide our final codebooks for each question on OSF (available here: https://osf.io/dfpj7/).

## 3. Results

First, we report thematic analyses of the two open-ended questions capturing participants’ perceptions of implicit bias interventions. Then, we summarize participants’ responses to Likert-style questions that followed each open-ended question, which largely validate the themes that emerged in these thematic analyses. Finally, we explore which individual differences predict participants’ support for implicit bias interventions.

### 3.1. Open-Ended Responses

#### 3.1.1. General Perceptions of Implicit Bias Interventions

All participants (*N* = 1016) provided their open-ended, general impressions of implicit bias interventions (i.e., “What do you think motivated the courts to adopt these interventions? What are your opinions and thoughts about the use of implicit bias interventions in the courts?”). Overall, participants’ responses were quite detailed (word count: *M* = 52.61, *median* = 46, *range* = 3–295). Notably, 36% of participants did not watch an implicit bias intervention prior to providing their perceptions and instead relied on a brief description of these interventions.^4^

OpenAI (2024) identified initial themes in the data relating to, for example, participants’ perceptions of the courts’ motivation (e.g., knowledge of historical inequities) and participants’ concerns about the interventions (e.g., whether the interventions are practical or just symbolic). From there, two coders edited and expanded these categories prior to and during the coding process. The final codebook included four major themes (see Table A1).

**Court Motivation.** When considering the motivation behind courts’ adoption of implicit bias interventions, participants commonly expressed the view that the courts were making a genuine effort to uphold impartiality and fairness across cases (55.4%, *n* = 563; i.e., *trial fairness*). The interventions were often discussed by participants as a proactive measure to address bias, while other times, as a reactive measure to address existing prejudice against specific groups.

Participants frequently contextualized the need to promote fairness by citing historical inequities in the U.S. or referring to group-based disparities in various outcomes (10.5%, *n* = 107; i.e., *group-based disparities*). Specifically, these participants often pointed out racial disparities at various stages of the criminal justice system (e.g., “racial profiling built into police training,” “unfair sentencing for people of color,” “the amount of innocent African Americans that are in prison”). Other times, they made general references to systemic bias or discrimination between groups.

Participants often suggested that the interventions served to remind jurors that they must judge cases with fairness and impartiality or reinforce the need to take their job seriously (19.1%; *n* = 194, i.e., *juror duties*). For instance, one participant described the intervention as a reminder that “prejudices should be abandoned before making important decisions in a trial.”

Others specifically discussed the educational value of implicit bias interventions (41.1%; *n* = 418, i.e., *bias education*). The manner in which participants discussed this varied widely. Many stated that they were not previously aware that they had biases—let alone how those biases might affect their decisions—and saw the interventions as a necessary first step for further corrective action. Others saw the interventions as a tool for encouraging reflection among jurors (e.g., “…I had never thought about unconscious biases until today. I have served as a juror on a criminal case and I would have liked to have heard this”).

Some participants also referenced the idea that everyone holds implicit biases, which is emphasized in the interventions. Specifically, 12.6% of participants (*n* = 128) described bias as universal (i.e., *everyone is biased*). In fact, many expressed support for the interventions precisely because they are so widespread. For instance, one participant noted that, “[b]y bringing awareness to this very normal, natural tendency we all have, judges and court officials can help mitigate the potential damage [from] jurors not recognizing why they think or believe a certain way.”

Some participants mentioned appreciating the reminder that, even if biases are inevitable, jurors have the responsibility to minimize their impact—whereas others were skeptical of the ability of the intervention to make an impact for this same reason. For example, one participant noted the following:

… we all have biases and that we need to recognize that they might interfere with our ability to render a fair verdict. I question how effective [implicit bias interventions] are. I think some bias is so deeply embedded in people that it is hard for them to recognize or care to.

Though very rarely, a few participants pushed back against the idea that implicit bias is universal (e.g., “… I do have problems with the idea that everyone has built in bias”; “I feel that basically people try to be fair at heart and that not everyone has prejudice thoughts”).

A few remaining subthemes emerged—albeit with low frequency—that offer unique insights into participants’ perceptions of the interventions and their beliefs about the court’s decision to adopt them. For brevity, we describe these low-frequency subthemes in the Supplemental Materials (pp. 16–18) and summarize them here. Though relatively rare, some suggested that the interventions were designed to help increase the accuracy of verdicts (3.3%, *n* = 34; i.e., *verdict accuracy*), often referencing the goal of reducing wrongful convictions and/or preventing guilty individuals from going free. Others speculated that the interventions were adopted to promote the public’s trust and confidence in the courts (3.1%; *n* = 31; i.e., *trust in courts*), to serve as a tool to assist in seating an impartial jury (2.2%; *n* = 22; i.e., *jury selection*), or to prevent litigants from challenging a verdict that is unfavorable to them (2.0%; *n* = 20; i.e., *satisfying litigants*). Though relatively more infrequent (i.e., 5–10 responses), some participants speculated that the interventions were in response to political polarization and/or the biasing impact of social media, whereas others framed the interventions as simply reflecting the broader cultural shift toward discussing bias across industries.

**Target(s) of Bias.** As participants considered the motivation for these interventions, we were also curious about what kinds of biases or group identities were particularly salient for them. We coded participants’ references to group identities when they directly named a group (e.g., “socioeconomic status”), described bias toward a group (e.g., “homophobia”), or referred to specific types of people within a social group (e.g., “Black people”). In total, 286 participants referenced at least one group identity (28.1%), typically by discussing their awareness of bias toward members of that group or the need for jurors not to judge litigants based on that particular group identity.

***Race and Ethnicity.*** The most commonly referenced target of bias, by far, was race and/or ethnicity (25.4%, *n* = 258). Many discussed the role of racial bias in court settings specifically (i.e., *racial bias in the courts*; 11.7%, *n* = 119), rather than racism more broadly. Others cited historical injustices or statistics that demonstrate racial disparities generally or in specific contexts (i.e., *systemic racism*; 5.9%, *n* = 60). Some participants identified these interventions as a product of “recent racially charged movements” like Black Lives Matter. Participants rarely criticized the courts’ focus on race-related issues (i.e., *critical of focus on race*; 1.3%, *n* = 13). A central theme among these few participants was the denial of racial bias. These participants often framed themselves as objective, morally driven individuals who can judge a case solely based on evidence (e.g., “color of skin doesn’t matter”; “there is no implicit bias in people surrounding race”). Others argued that the court’s focus on racial bias is divisive and politically motivated. We describe related concepts in depth under “Defensive Response” below.

Because judging a case with a Black (vs. White) litigant can increase the salience of race (Salerno et al., 2023), we also investigated whether the likelihood of bringing up race/ethnicity differed for those who judged a Black versus White plaintiff in the broader experiment. While mentions of race and/or ethnicity were higher among participants judging a Black plaintiff (30.0%, *n* = 152) relative to those judging a case with a White plaintiff (20.4%, *n* = 104), *χ*^2^(1, *N* = 256) = 11.78, *p* < 0.001, we nonetheless found that participants in the White plaintiff condition still mentioned race frequently—and were twice as likely to mention race as the next frequently mentioned group identity (i.e., gender; 10.4%, *n* = 53).

***Other Group Identities.*** Beyond race, participants believed that implicit bias interventions could help many different kinds of groups in the courtroom. Participants referenced gender (10.5%, *n* = 107), age (3.2%, *n* = 33), socioeconomic status (3.1%, *n* = 32), physical appearance (2.8%, *n* = 28), religious identity (2.4%, *n* = 24), and LGBTQ+ identity, which included both gender minorities (e.g., nonbinary individuals) and sexual minorities (2.3%, *n* = 23).

Some participants reflected on the importance of group identities through the lens of counterfactual thinking—an anti-bias strategy that is often referenced in the interventions. For example, one participant in the judicial instructions condition noted that the judge’s recommendation to consider how they might feel differently if a person were in another demographic group (e.g., race, religion, sex) “was a good reminder and effective process to be sure that we were not being influenced subconsciously.”

**Concerns and Criticisms.** It is critical to examine participants’ concerns about the interventions because they may impact their capacity to be effective. We found that one out of six participants (16.2%; *n* = 165) expressed some concern about or criticism of implicit bias interventions. Three major subthemes emerged.

First, some participants questioned the interventions’ effectiveness (i.e., *questioning effectiveness*; 8.8%, *n* = 89). They raised doubts about whether implicit bias interventions are able to mitigate or eliminate juror bias, often grappling with the ideas that biases are resistant to change, innate, and/or pervasive. Further, some participants critiqued the interventions’ ability to be effective based on the premise that implicit biases, by definition, operate outside a person’s awareness. Many participants stressed that the interventions were too “brief” or “short” to be effective, referring to implicit bias interventions as a “good start” but ultimately insufficient. One participant, for example, noted that addressing bias “requires consistent intervention (rather than just one video) because these implicit biases are hard to break.” Despite doubts about the interventions’ effectiveness, more than half of these participants (*n* = 45) framed their responses in a way that expressed support for their use (e.g., “it’s worth a shot”). This alludes to the point that people often support interventions that lack empirical support, which we explore in depth in the discussion.

Second, some participants discussed potential unintended consequences of the interventions (i.e., *unintended consequences*; 3.2%, *n* = 33). Participants noted that the interventions might make it more challenging for jurors to reach their verdicts, “complicate the deliberation process,” and “make [jurors] think they are possibly biased when in fact they may not be.” These participants also questioned whether the interventions might lead jurors to “overcompensate” or “overcorrect” for their biases, potentially leading them to be too lenient on minority group members and too harsh on majority group members. On the other hand, some participants expressed concern that these interventions could trigger a backlash effect among certain jurors, suggesting that some may intentionally “ramp up any bias they have in defiance” of the intervention. Notably, though, our broader experiment did not find evidence of a backfiring effect among mock jurors’ verdicts (Lawrence et al., 2025).

Third, a minority of participants rejected the premise of the interventions or the interventions themselves (i.e., *defensive response*; 2.5%, *n* = 25). These participants often framed the interventions as offensive, manipulative, or politically motivated. Some described the interventions as divisive (e.g., using “divisive dialogue that further alienates people in this country”). Others critiqued the interventions by suggesting that bias is exaggerated, not “as prevalent as people are led to believe,” and overstated by the media. Relatedly, some claimed that jurors are already motivated to act without bias, making these types of interventions unnecessary.

#### 3.1.2. Evaluations of Specific Interventions

Participants who were randomly assigned to watch an intervention video—either a standard educational video about implicit bias or judicial instructions about implicit bias—responded to the following: “In a few sentences, what was your impression of this video? What did you learn or take away from the video?” Three participants in the intervention conditions did not respond, leaving 318 participants in the educational video condition and 328 in the judicial instructions condition in the following analyses. Again, participants’ responses were generally detailed (word count: *M* = 40.30, *median* = 35, *range* = 4–506). Using OpenAI’s (2024) ChatGPT-4o, we identified four common themes in participants’ responses (content takeaway, educational value, personal reflection, and impression of overall video/video features). We then edited and expanded these categories and identified 18 unique subthemes in participants’ responses (see Table A2).

**Content Takeaway.** Just over half of participant responses (54%; *n* = 349) referenced at least one of the four subthemes describing takeaways from the content presented in the videos. Approximately one-third of participants (30.3%; *n* = 196) shared some information they had learned about implicit bias, whether accurate or not (e.g., that it is unconscious or universal; i.e., *conceptual understanding of implicit bias*). About 18% of participants’ responses (*n* = 120) discussed how the videos reinforced the importance of both understanding and being aware of biases (i.e., *importance of bias awareness or reflection*). About a quarter of participants’ responses (25.9%; *n* = 167) noted that the video informed them of their duties as a juror (e.g., “I learned that if you are feeling biased for any reason that you should express this to the lawyers and they can decide if or how it might affect the case”; i.e., *duties of a juror*). Rarely (2.5%, *n* = 16), participants mentioned taking away that these videos were intentional, systemic efforts by the legal system to address bias and inequality (i.e., *systemic efforts of the justice system towards fairness*).

Given that the educational videos and judicial instructions differed somewhat in their content, we explored differences in participants’ takeaways, depending on the specific type of intervention video they watched. While participants’ reference to the importance of bias awareness, *p* = 1.00, and systemic efforts for fairness in the justice system, *p* = 0.62, did not differ based on intervention condition, participants who watched the educational videos were more likely to share in their responses a conceptual understanding of what implicit bias is and/or how it operates (38.1%, *n* = 121) compared to those who watched the judicial instructions video (*n* = 75, 22.9%), χ^2^(1, *N* = 196) = 10.80, *p* = 0.001. For example, one participant noted the following:

…[the educational video] made me understand exactly what implicit bias was, and how we may all have it, despite our best efforts. The part about how our unconscious brain works made a lot of sense to me, and is something I will take with me in the future.

However, participants were more likely to reference taking away an understanding of their role and duties as a juror after watching a video of judicial instructions about bias (*n* = 117, 35.7%) compared to those who watched an educational video about bias (*n* = 50, 15.7%), *χ*^2^(1, *N* = 167) = 26.88, *p* < 0.001. Perhaps this is because jurors who viewed the judicial instructions video were informed of their expectation of being unbiased by the same judge who delivered other instructions in the mock case (e.g., liability and damage awards instructions), making their duties particularly salient. In contrast, the educational videos presented a more general explanation of implicit bias and how to apply it but did not include the same judge that they saw in their specific case.

**Educational Value.** Approximately one-third (30%; *n* = 194) of participants who were randomly assigned to watch an implicit bias intervention reported learning something new from the intervention (i.e., *new concepts*), with one participant responding as follows:

[This video] had a huge impact on my understanding of implicit bias. I learned that as a juror I must be aware of my unconscious bias and that it can happen quickly and automatically without much thought or realization. I also learned to stop and think about my decisions before settling on my final thoughts. Lastly, I’ve learned that it would be wise to dive into my subconscious and think about whether a person’s age, gender, or their race had possibly influenced my decisions.

Less frequently, participants acknowledged that they already were aware of the content but appreciated the video as a reminder for themselves (7.3%; *n* = 47; i.e., *prior knowledge but appreciated reinforcement for self*) or for others (1.4%; *n* = 9; i.e., *prior knowledge but appreciated reinforcement for others*).

Additionally, some participants (8.8%; *n* = 57) reported concerns or critiques about the educational value of these videos (i.e., *concerns or critiques about the educational value*), suggesting that they were too surface level or commonsense (e.g., “I don’t really feel like I learned anything new, this stuff is pretty well covered in college”), lacked practical applications (e.g., “I can understand taking a moment to focus on the evidence and making sure you’re not focused on your own biases and stereotypes, but how exactly can you be absolutely sure you’re not doing this?”), or were inaccurate or in the service of a political agenda (e.g., “I learned that a bunch of anecdotal stories that probably aren’t even true are what passes for science to these types. Essentially whatever confirms their beliefs is science and everything that doesn’t is misinformation”).

Participants who were in the educational video condition (35.5%, *n* = 113) were much more likely to report learning something new than those in the judicial instruction condition (24.7%, *n* = 81), χ^2^(1, *N* = 194) = 5.28, *p* = 0.02. This may reflect that the educational videos tended to be more in-depth and more likely to use real-world examples from basic social psychology than the judicial instructions videos. There were no other differences related to participants’ discussion of educational value between the intervention conditions.

**Personal Reflection.** Despite many participants acknowledging their educational value, few participants mentioned applying the information to their own lives. In fact, participants rarely mentioned that these videos made them more aware of their own personal biases (4.6%; *n* = 30; i.e., *personal bias awareness*) or encouraging them to make changes to the way they express these biases (2.8%; *n* = 18; *personal change or action regarding bias*). The likelihood of mentioning these personal reflections about bias did not differ based on the type of video that the participant watched (i.e., educational video vs. judicial instructions), *p* = 0.49.

**Video Features.** Over half of participants (52.5%; *n* = 339) mentioned a positive impression of the video that they watched, either through a general remark about the video (e.g., “It was a good video”; i.e., *overall*) or by noting a specific feature of the video that they liked (i.e., *delivery*, *clarity of content*, *content*, *length/pacing*, *examples*, *speakers*, *strategies*, *tone*). Far fewer participants reported mixed (14.4%; *n* = 93) or negative (6.0%; *n* = 39) impressions of the videos. Those with mixed opinions often remarked on a single negative feature of the video but included praise for other features or the video as a whole (e.g., “I liked the video, the presenters were easy to understand and acted professionally. It was a bit too long and repetitive. It would help people think about their biases”). Conversely, those with negative opinions often criticized the video as a whole, rarely identifying specific features in their responses (e.g., “It reminded me of DEI brainwashing, and I actively rejected it. It was disgusting and made to push an agenda”). Overall, participants who watched an educational video (*n* = 190, 59.7%) were more likely to mention a positive impression than participants who watched a judicial instruction video (*n* = 149, 45.4%), χ^2^(3, *N* = 646) = 16.11, *p* = 0.001.

Participants were much less likely to remark about the specific features of the video compared to reporting their overall impression. Participants who watched an educational video were more likely to comment that the intervention was engaging (e.g., had high production value, was interesting and kept their attention, was well made; 30.2%, *n* = 96; i.e., *delivery*), clear (e.g., understandable, easy to follow; 17.0%, *n* = 54; i.e., *clarity of content*), and included good examples (e.g., examples helped them understand the content better, liked the scope of examples provided; 13.5%, *n* = 43; i.e., *examples*) compared to those who watched the judicial instructions video (*delivery*, 6.1%, *n* = 20; *clarity of content*, 12.2%, *n* = 40; *examples*, 1.2%, *n* = 4). Despite overall higher levels of positive impressions toward the educational video, participants who watched a judicial instruction video were more likely to comment that these videos were of an appropriate length (4.9%, *n* = 16; i.e., *length/pacing*) compared to those who watched an educational video (1.6%, *n* = 5), which some participants claimed were too long or repetitive. There were no differences across intervention conditions in participants’ references to the quality of content presented (e.g., how informative or useful the content was; i.e., *content*), speaker selection (e.g., the choice of speakers based on things like diversity or position in the court; i.e., *speakers*), the inclusion of practical strategies (i.e., *strategies*), or tone (e.g., generally non-defensive or approachable; i.e., *tone*).

### 3.2. Quantitative Ratings

Following these open-ended responses, we also assessed participants’ perceptions by directly asking them to rate implicit bias interventions on a variety of dimensions. First, participants answered eight questions about reasons they might support or not support implicit bias interventions. On average, participants agreed with items reflecting support and disagreed with items reflecting a lack of support (see Table A3).

Participants then explicitly stated whether they supported or opposed the use of implicit bias interventions in real trials. The overwhelming majority of participants (91.2%, *n* = 927) expressed some level of support for implicit bias interventions, with most participants either somewhat supporting (14.7%, *n* = 149), supporting (38.1%, *n* = 387), or strongly supporting (38.5%, *n* = 391) their use in real trials. Relatively few participants (8.8%, *n* = 89) expressed any level of disapproval of implicit bias interventions in real trials.

Notably, participants’ implicit bias intervention condition influenced their support. Participants who watched an implicit bias intervention expressed greater support for their use in courts relative to those who did not, whether it was delivered via an educational video (vs. no intervention), *B* = 0.31, *SE* = 0.09, *p* < 0.001, or via judicial instructions (vs. no intervention), *B* = 0.30, *SE* = 0.09, *p* < 0.001. Participants in the two intervention conditions expressed similar levels of support for implicit bias interventions, *B* = 0.01, *SE* = 0.09, *p* = 0.95.

In addition, we asked participants who were randomly assigned to an implicit bias intervention condition (educational video, *n* = 319; judicial instructions, *n* = 330) to rate the intervention they watched on a variety of dimensions following their open-ended impressions of the intervention. Specifically, on a 5-point scale, they indicated the extent to which they agreed that the intervention they watched was *helpful*, *engaging*, *informative*, *confusing*, *misguided*, and *unscientific*. Mirroring the thematic analysis, participants had generally positive impressions of the specific video they watched (descriptive statistics and *t*-statistics reported in Table A4). On average, participants agreed with items reflecting positive attributes (e.g., helpful, informative) and disagreed with items reflecting negative attributes (e.g., confusing, misguided). Participants who watched the educational video reported that the intervention was more informative and engaging than participants who watched the judicial instructions. However, there were no differences in perceptions of these types of intervention as helpful, confusing, unscientific, or misguided.

### 3.3. Individual Differences in Support for Implicit Bias Interventions

We also investigated which individual difference measures were associated with participants’ support for implicit bias interventions (see Table A5). Consistent with our hypotheses, participants’ political conservatism, psychological reactance, skepticism of social scientists studying race, and endorsement of select advantaged-group member strategies (i.e., *deny*, *defend*, *distance*) were associated with significantly less support for implicit bias interventions in courts. Participants’ endorsement of the *dismantle* advantaged-group member strategy was significantly associated with greater support. Although we did not preregister any hypotheses about the relationship between participants’ racial biases and their support for implicit bias interventions, higher explicit bias scores were significantly associated with less support, but implicit bias scores were unrelated to support.

To determine which of these variables are uniquely predictive of participants’ support for implicit bias interventions, we entered our preregistered individual difference measures—political conservatism, psychological reactance, skepticism of social scientists studying race, and each advantaged-group member strategy—into a simultaneous linear regression (Table A6). These predictors accounted for 56% of the variance in participants’ support for implicit bias interventions. In the simultaneous regression model, participants’ political conservatism, psychological reactance, and skepticism of social scientists studying race significantly predicted lesser support for implicit bias interventions, and participants’ endorsement of the *dismantle* advantaged-group member strategy significantly predicted greater support for implicit bias interventions.

Because of our key interest in political orientation as a predictor of participants’ perceptions, we explored this variable in greater detail. First, although both conservative and liberal participants, on average, expressed support for implicit bias interventions, liberal and liberal-leaning participants (*M* = 5.37, *SD* = 0.76) were more supportive of implicit bias interventions than were conservative and conservative-leaning participants (*M* = 4.55, *SD* = 1.37), *t*(1015) = 11.94, *p* < 0.001, *d* = 0.75. Relatedly, for items related to reasons one might support or not support implicit bias interventions, conservative and conservative-leaning participants were generally more likely to disagree with positive items and agree with negative items about implicit bias interventions than liberal and liberal-leaning participants (see Table A3).

## 4. Discussion

It is critical to investigate the impact of interventions that aim to address inequalities in a timely manner (Najdowski & Stevenson, 2022), particularly when an intervention is newly developed (Gegenfurtner et al., 2009). In a previously published experiment, we found little evidence that implicit bias interventions influenced participants’ decisions in a mock civil trial (Lawrence et al., 2025). Here, we examined a second question about participants’ *perceptions* of the interventions to investigate whether the interventions may, in part, have limited effectiveness because participants reject or dislike them. However, we largely did not find support for this explanation: Only a small, but vocal, minority expressed defensiveness or distaste for the interventions. Participants, all of whom self-identified as White, broadly supported the use of implicit bias interventions in courts, both in direct survey responses and open-ended responses. When asked why they believed courts implemented these interventions, participants most often cited a sincere effort to ensure fairness and acknowledged the value of bias education. Many appeared to resonate with the idea that bias is a universal and natural human tendency.

Participants frequently referenced race and ethnicity when discussing groups that could benefit from these interventions, but they also recognized that litigants may face bias based on gender, age, socioeconomic status, religious identity, and LGBTQ+ identity, for example. When asked more directly about the interventions’ impact, participants generally agreed that implicit bias interventions make trials fairer and demonstrate that the courts care about impartiality.

Despite this broad support, some individual difference measures predicted relatively greater or lesser support for the interventions. Specifically, political conservatism, psychological reactance, and skepticism toward social scientists studying race significantly predicted less support for implicit bias interventions, and endorsement of the *dismantle* advantaged-group member strategy significantly predicted greater support. Participants’ responses also reflected critical engagement with the topic. Some questioned the interventions’ effectiveness, noting the challenge of addressing unconscious biases. Others worried about unintended consequences, such as jurors “overcorrecting” for potential biases or backlash from jurors who might view the interventions as manipulative or divisive. However, only a small minority criticized the motives behind the interventions or framed them as accusatory or offensive. Notably, such criticisms may be relatively more widespread in the recent political landscape, which we explore in greater detail later.

### 4.1. Comparing Perceptions of Judicial Instructions Versus Educational Videos

This study also gave us the opportunity to assess laypeople’s perceptions of implicit bias interventions in their two most common formats: an educational video and instructions delivered by a judge (Kirshenbaum & Miller, 2021). Participants generally viewed both kinds of interventions quite positively and as similarly helpful—though there appeared to be some key differences in what they viewed these videos as helpful for. For instance, participants who saw an educational video more often noted that these videos were engaging and did a good job conveying what implicit bias *was*, whereas participants who viewed a judicial instructions video were more likely to note that these videos do a good job of explaining what jurors should *do* regarding implicit bias. While we argue that there is not currently enough evidence of the interventions’ effectiveness to support their use—particularly in the absence of other empirically-based strategies that improve trial fairness—we identified a few considerations for courts interested in educating jurors about implicit bias, including benefits and costs to each intervention format.

Educational videos may better capture jurors’ attention relative to judicial instructions, as participants who watched an educational video rated their intervention as more engaging and informative than participants who watched the judicial instructions intervention. This sentiment was echoed in their open-ended impressions of the videos: Participants who saw an educational video were more likely to report that these videos were delivered well, were clear in their content, had useful examples, and taught them something new relative to those who watched a judicial instructions video. One participant went so far as to write the following:

I think that a well produced video on implicit bias is far more effective than a judge reading from a piece of paper. People are far less inclined to pay attention to someone dryly reading from a paper than they are to a well-produced video with animations.

This aligns with research in educational psychology, which demonstrates that combining visual and auditory elements in an intervention can promote learning more effectively than auditory information alone (Baggett, 1984). To reiterate, however, even if jurors *think* they learn more from the education videos, this may not be sufficient to meaningfully influence their behavior.

That said, judicial instructions may offer their own benefits over other forms of implicit bias education. As reported in our prior article (Lawrence et al., 2025), participants—all of whom self-identified as White—awarded more favorable verdicts to Black plaintiffs when they received judicial instructions about implicit bias relative to those who received only standard judicial instructions. However, this was not observed among those who watched an educational video about implicit bias. One explanation for this may be that jurors take instructions more seriously when they come from a judge, rather than a routine educational video, because they rely on the judge to help them perform their duties in that specific case (Diamond & Hans, 2023; Marder, 2022). We found some support for this idea: Participants who watched a judicial instructions video were more likely to reference their duties as a juror in their open-ended responses than those who watched an educational video. Prior research suggests that people’s motivation to engage with interventions depends on whether they believe the intervention is relevant for their role (Gegenfurtner et al., 2009). Perhaps judicial instructions about implicit bias feel more relevant and important to a juror’s role than educational videos. Future research could explore whether jurors feel more motivated to disclose and monitor their own biases if a judge reinforces their obligations during judicial instructions.

### 4.2. Conservative Support Amid DEI Backlash

The infrequency of defensive or negative responses to implicit bias interventions in this study, particularly among political conservatives, may be seen as surprising. While we found support for our hypothesis that political conservatism was associated with less support for implicit bias interventions in courts, most conservatives (83%) still expressed some level of support for them. This stands out from the broader trends of political divisiveness around DEI initiatives (Minkin, 2024; Palmer, 2024; Sheen, 2023) and raises the following question: Why did conservatives show relatively high support for implicit bias interventions in courts when their support for other kinds of DEI initiatives tends to be relatively low (Brodbeck et al., 2025; Minkin, 2024)?

One explanation may be that, despite political differences, Americans share many core values. For example, about 90% of both Democrats and Republicans believe that fairness is very or extremely important (Klepper, 2023). Recent survey data finds that eight in ten Americans somewhat or strongly support principles underlying DEI practices (e.g., including people of all backgrounds when discussing the state of our nation), despite polarization in responses to the term “DEI” (Brodbeck et al., 2025). We may not have triggered these polarized responses, in part, because we did not use the term DEI anywhere in our survey.

However, Americans across political lines may have different opinions about *how* to achieve fairness. Democrats tend to be more concerned with how historically marginalized groups are treated (e.g., by the justice system; Dunn, 2020) and tend to be more supportive of targeted interventions like quotas that aim to uplift underrepresented groups (Goode, 2024). In contrast, implicit bias interventions in courts may be less divisive because they are framed in more universal terms. They tend to emphasize the need to be fair and impartial to all litigants, rather than supporting specific groups. In fact, some participants noted that their support of implicit bias interventions depended on whether they are used consistently for litigants of all backgrounds. For example, one participant wrote:

I think the idea of courts reminding jurors of implicit bias is a good idea only if the bias instructions refer to ALL people, not just blacks, Muslims, Hispanic, etc. I believe everyone has some type of bias in their hearts and minds. So, if the instructions about implicit bias don’t include all races, the situation actually becomes worse not better.

That being said, another explanation for the relatively high support of implicit bias interventions among conservatives and liberals alike may be that these evaluations were influenced by response biases, such as social desirability effects or demand characteristics. We continue this discussion in Section 4.4, where we discuss the likelihood of response biases in our survey and the extent to which these may still be a threat amidst changes in social norms and in the political climate where DEI is increasingly scrutinized.

### 4.3. The Benefits—And Costs—Of Improving Perceptions of Implicit Bias Interventions

It is important not to conflate jurors’ favorable impressions concerning the effectiveness of these interventions with their *actual* effectiveness in reducing biased verdicts. As we discussed, whether implicit bias interventions actually improve trial fairness remains a largely open question: Interventions that might be practical for courtroom settings generally fail to meaningfully reduce people’s biases (Lai et al., 2014, 2016). This literature is in line with our experimental findings that neither educational videos nor judicial instructions mitigated the link between White mock jurors’ explicit racial biases and their verdicts for Black plaintiffs (Lawrence et al., 2025). Importantly, though, our mock juror study did offer preliminary evidence that judicial instructions—but not educational videos—might improve outcomes for Black plaintiffs, regardless of jurors’ bias level (Lawrence et al., 2025)—a finding that needs replication and further investigation before courts rely on this intervention. While these interventions may indirectly enhance trial fairness by influencing juror behavior during voir dire or deliberations, this has yet to be thoroughly explored (for an exception, see Lynch et al., 2022).

It is quite common that people support interventions that *feel* effective, even when evidence suggests otherwise. Many widely endorsed policies targeting justice issues have failed to deliver on their promises. For example, education-based programs aimed at protecting at-risk youth, like the popular D.A.R.E.E. program, have not been shown to reduce drug use (Singh et al., 2011). Similarly, “scared straight” programs ironically increase the likelihood that at-risk youths will offend (Petrosino et al., 2013), and “tough on crime” sentencing fails to reduce crime rates (Nelson et al., 2023). Sex offender registries, which are often seen as essential for community safety, do not deter reoffending and may even increase the risk of future offenses (Covert, 2023; Letourneau et al., 2015). These examples reflect a broader pattern: Interventions that align with our intuitions often receive strong public support, even when empirical evidence does not demonstrate their effectiveness. This same dynamic may be seen in the legal system’s growing embrace of implicit bias interventions.

Our findings suggest that implicit bias interventions may help the courts appear more procedurally just. Participants, on average, believed that implicit bias interventions make trials fairer and demonstrate that the courts care about impartiality. Decades of research on procedural justice theory has demonstrated the benefits of improving perceptions of institutions (Tyler, 2003). Specifically, when people perceive institutional procedures as fair, they are more likely to show compliance with those institutions (Tyler, 2007). Let us apply this to the courts: a litigant who receives an unfavorable ruling—such as a business owner who loses a contract dispute—may be more accepting of an outcome and more likely to follow the court’s orders if they believe the court process that led to that outcome was fair. Our findings suggest that the use of implicit bias interventions in courts may increase that perception of institutional fairness.

Unfortunately, there is not yet compelling evidence that implicit bias interventions actually work. In light of this, there may be downsides to inflating perceptions of fairness for a system that might continue to produce unfair outcomes. In the context of police–citizen interactions, some scholars have expressed concern that improving perceptions of an unjust institution—in this case, policing—may veil systemic issues or inequalities which, in turn, can get in the way of additional reform efforts (e.g., Fields, 2023; Schaap & Saarikkomäki, 2022). If the public believes that court procedures are fair, and the courts feel they have done their due diligence to address documented disparities in trial outcomes, they may not adopt additional strategies to improve trial fairness that may be more effective. Another potential downside to improving perceptions of the courts’ fairness is that it could make the appeals process more challenging for litigants. If an individual did not get a fair trial, they may have less success appealing their case because attorneys can point to the court’s debiasing efforts via an implicit bias intervention. This highlights the urgent need to prioritize research that determines the effectiveness of the interventions and whether improved perceptions of court fairness are warranted.

As social scientists work to determine what effects implicit bias interventions actually have in jury selection, deliberations, and trial outcomes, courts that use the interventions must pair them with other empirically supported efforts (Elek & Miller, 2021). For instance, previous work has found that asking specific, attitude-based questions during voir dire is more helpful in evaluating which jurors should be dismissed than general questions alone (Salerno et al., 2021). Thus, extending the voir dire process to give attorneys enough time to assess prospective jurors’ biases may be valuable. Furthermore, diverse groups—in terms of attitudes, experiences, and demographics—make better decisions (for a review, see Sommers & Norton, 2008). In legal settings, specifically, research has shown that racially diverse juries make fewer errors and discuss more case facts than all-White juries (e.g., Bergold & Kovera, 2022; Sommers, 2006). Courts must eliminate barriers to representative juries.

In sum, the use of implicit bias interventions is a meaningful demonstration of the court’s commitment to upholding trial fairness. It garners significant lay support across the political spectrum. Yet, because we do not yet have consistent evidence of their effectiveness, courts must harness other strategies to build greater confidence in their efforts to ensure fair trials.

### 4.4. Limitations and Future Directions

Some characteristics of our study may limit the generalizability of our results. First, as is the case with any mock juror study, it is reasonable to question whether participants’ perceptions of the implicit bias interventions would generalize to real jurors. Although participants provided detailed and thoughtful open-ended responses, suggesting that they took their task seriously—and prior work suggests no consistent differences between simulated and real trials (Bornstein & McCabe, 2005)—laboratory settings always lack the stakes of real trials. At the same time, we took several steps to enhance external validity (Bornstein et al., 2017) by providing detailed trial stimuli and thorough judicial instructions (see Lawrence et al., 2025). Because participants rated the interventions in the context of a mock case, this better mirrored how real jurors would encounter them and may have prompted participants to reflect on how the interventions might shape their thoughts and behaviors in a real case. Nevertheless, future research should assess real jurors’ perceptions.

Second, participants’ evaluations of the interventions may have been shaped by features of the specific mock case that they judged. For example, one participant referenced specific characteristics of the plaintiff’s appearance and lifestyle choices that “created a possible unfair picture…of [the plaintiff’s] trustworthiness” when justifying their support for the interventions. Fortunately, we did not find evidence that the manipulations substantially impacted our measures. Judging a Black (vs. White) plaintiff did not affect participants’ evaluations of the interventions nor their responses on the individual difference measures (as detailed in the Supplemental Materials)—suggesting these evaluations and measures were capturing something relatively stable.

However, the extent to which jurors believe that the interventions are relevant and useful may depend on specific case features, including the ambiguity of case facts, characteristics of litigants, and whether they judge a case in a civil or criminal context. A promising direction for future research, for example, is to test the interventions with litigants from other marginalized groups who may be targeted by juror bias (e.g., based on disability, socioeconomic status, or LGBTQ+ identity). Further, our results might not generalize to a situation where jurors are asked about their attitudes before judging the case. Future research should counterbalance these individual difference measures—such that they are captured either before or after the intervention evaluations—to allow for a concrete test of the intervention without potential order effects.

Third, participants’ evaluations may have been influenced by features of the specific intervention they watched. To address this, we used stimulus sampling (Wells & Windschitl, 1999), such that mock jurors in the intervention conditions watched one of eight possible interventions. Notably, though, participants who were randomly assigned to the judicial instructions condition all viewed implicit bias instructions read by the same White female mock judge. Because some participants noted that her delivery was “robotic” and lacked “care or concern,” participants may have found the judicial instructions videos more engaging if her delivery was more dynamic. Future research should explore whether the current findings extend to judicial instructions read by different judges.

Our sampling approach also constrained potential generalizability. We targeted a sample of community participants who self-identified as White. This choice was strategic. In our broader experiment, we aimed to increase the likelihood that we would detect a bias effect in participants’ case decisions that reflected real-world data—specifically, an anti-Black bias effect—which would allow for a more effective test of interventions designed to reduce that bias. Importantly, attitudes and responses of this group may not reflect those of people of color. For instance, though defensive reactions were relatively rare for our White sample, they may be even less common among people of color. Relative to White people, people of color tend to have more favorable perceptions of DEI initiatives (e.g., Bowman, 2025; Gündemir et al., 2024) and perceive lower levels of fairness in court outcomes and procedures (Rottman & Hansen, 2000; Sun & Wu, 2006). Relatedly, certain psychological factors might predict support differently across racial groups. Prioritizing inclusive and diverse sample recruitment, when possible, is critical—especially with regard to participant race, as people of color are often underrepresented among participant pools in psychological research (Torrez et al., 2022). A critical next step is to investigate how various intersectional identities—including, but not limited to, one’s racial group—shape perceptions of and behavioral responses to implicit bias interventions. Further, specialized implicit bias content and toolkits have been developed for judges, prosecutors, and public defenders (American Bar Association, 2016). Because these groups substantially impact court outcomes and are also vulnerable to making biased decisions (Sommers & Marotta, 2014; Wistrich & Rachlinski, 2017), it would be valuable to explore their perceptions of these interventions as well (for preliminary research on judges’ perceptions, see Giannetta et al., 2023; Kirshenbaum & Miller, 2021).

Finally, because implicit bias interventions emphasize fairness and equality, participants who are skeptical of them—for myriad reasons—may have felt social pressure to keep their concerns private. Social desirability bias, which is a common threat to survey responses on morally charged topics (Tourangeau & Yan, 2007), has been documented in participants’ responses about DEI initiatives (Boring & Delfgaauw, 2024) and in studies that manipulate race (e.g., Salerno et al., 2023). Other researchers have expressed concern about the role of social desirability in similar research (e.g., Isenberg & Brauer, 2024). Considering that participants’ opinions about the interventions did not have any direct consequences, they may have artificially inflated their positive responses. This response bias may have been exacerbated by demand characteristics. For example, if participants believed that we intended to promote the interventions in this study, they may have inflated their positive responses to align with this perceived goal.

Importantly, we see several reasons to be cautious in assuming that social desirability bias substantially altered our findings. First, we did not find evidence that the experimental manipulations meaningfully influenced participants’ responses. Second, we took steps to mitigate social desirability concerns. For example, we framed questions neutrally to demonstrate that there was not a normative response, such as in the prompt before the first intervention evaluation measure (“People have different opinions about why these implicit bias interventions are being used in courts. We’re interested in hearing your thoughts…”). We also reinforced anonymity and honesty in prompts before participants answered individual difference measures that may be particularly socially sensitive (e.g., “Any view is acceptable, so please answer openly and honestly. We simply want to better understand how you think about this issue. Your responses will be completely confidential”). We also observed variability in participants’ responses—including some participants’ clear criticism of bias education—which suggests that at least some participants were able to overcome any pressures related to social desirability bias. There are certainly other strategies that researchers could use to further mitigate social desirability concerns. For example, researchers could harness moral credentialing techniques (Monin & Miller, 2001), which have been applied to mock juror research (Salerno et al., 2023). If participants are able to demonstrate that they care about diversity or equity in some other way, it may credential them to share their more honest opinions about this socially sensitive topic.

Notably, the extent to which certain views on these topics are socially proscribed may have shifted since we collected our data in June of 2024. Although political attacks on DEI initiatives have existed for years (e.g., anti-DEI bills: Goldberg, 2024; Iyer & Boyette, 2023), they escalated significantly when Donald Trump signed an executive order terminating all government programs, policies, funding, and activities related to DEI approximately six months following our data collection (Trump, 2025). This executive order criticized research on diversity and inclusion and characterized DEI initiatives as “illegal and immoral.” Considering that people are often motivated to shift their own policy views to better align with elected politicians (Lenz, 2012), it is possible that negative opinions about implicit bias interventions have become more socially normative. It is also possible, however, that the glaring political polarization in the United States on this topic has reinforced the idea that bias-mitigating interventions are needed, and as a result, ironically *increased* bipartisan support for them, even if liberals and conservatives differ in whom they believe is most affected by group-based biases. It may be valuable to reassess laypeople’s perspectives in light of these recent political shifts.

## 5. Conclusions

United States courts have recently implemented interventions that educate jurors about implicit bias. We explored White participants’ perceptions of implicit bias interventions. Broadly speaking, participants supported the use of these interventions in courtrooms, though certain individual difference measures—including political conservatism, psychological reactance, and skepticism of social scientists studying race—predicted relatively less favorable perceptions of them. Open-ended and Likert-style responses regarding perceptions of these interventions were quite nuanced, including differences in how participants perceived the interventions based on their format (educational video, judicial instructions). For instance, participants perceived educational videos as relatively more informative and engaging, whereas participants who watched the judicial instructions about implicit bias more often referenced their duty as a juror to act without bias. These perceptions may influence the interventions’ ability to improve trial fairness as courts intend. Ultimately, however, conclusive evidence demonstrating whether they actually improve trial fairness is necessary to determine whether positive perceptions of the interventions are warranted, unwarranted, or perhaps even harmful.

## Data Availability

The data from this study is available on the Open Science Framework at https://osf.io/dfpj7/files/osfstorage, (Data > Study 2 Data).

## Notes

1 Although these manipulation checks were designed for the primary research question and were unrelated to the current research questions, we reasoned that our sample would be of higher quality if we excluded participants who showed signs of inattention to other aspects of the experiment.

2 We intentionally presented participants with two prompts (i.e., double-barreled questions) to encourage detailed, thorough responses. In some responses to this first open-ended question, whether participants’ statements reflected their personal beliefs or their perceptions of the court’s motivations for adopting the interventions required some interpretation. The coders made their best judgment about which part(s) of the question participants were responding to based on the full context of their response and resolved ambiguities via discussion. Although there was also some overlap in the topics covered in each open-ended prompt, we chose to analyze these responses separately because of differences in the specificity of the prompts.

3 Participants completed four general attention checks (e.g., “If you are paying attention, select [scale point]”), four content-specific attention checks (e.g., “What condition did [the plaintiff] develop as a result of his injury?”), and two manipulation checks, identifying which implicit bias intervention they received (if any) and selecting the correct plaintiff photograph among fillers. Participants were excluded if they answered either manipulation check incorrectly or if they incorrectly answered three out of four general attention checks or trial content attention checks.

4 Some participants who did not watch an intervention noted that this made it more challenging to provide their perspectives (e.g., “Without seeing an exact example of an implicit bias intervention I’m not sure if it would help me identify bias and be more objective or not”). However, their reactions are nonetheless valuable, considering that laypeople’s perceptions of the courts can be shaped by what they learn about court procedures without experiencing them firsthand. Consider, for example, that people tend to have strong opinions about DEI initiatives, despite the fact that, according to a national study, less than one-third report being at least somewhat familiar with them (vs. “not too familiar” or “not at all familiar”; Brodbeck, 2025). Capturing these data allowed us to compare the extent to which participants supported the interventions based on whether or not they were exposed to them.

## Supplementary Materials

The following supporting information can be downloaded at https://www.mdpi.com/article/10.3390/bs15091269/s1: Table S1: Content Comparison of the Implicit Bias Educational Videos (Stimulus-Sampled); Table S2: Content Comparison of the Judicial Instructions (Stimulus-Sampled); Table S3: Perceptions of Implicit Bias Interventions by Plaintiff Race Condition; Table S4: Individual Difference Measures by Plaintiff Race Condition; Table S5: Perceptions of Implicit Bias Interventions by Implicit Bias Intervention Condition; Table S6: Individual Difference Measures by Implicit Bias Intervention Condition; Table S7: AI-Generated Themes by Plaintiff Race Condition (Thematic Analysis 1); Table S8: Percent Agreement for Thematic Analysis 1; Table S9: Percent Agreement for Thematic Analysis 2.

## Appendix A

**Table A1:** General perceptions of implicit bias interventions’ themes and subthemes.

**Court Motivation (93.3%, *n* = 948)**	**Targets of Bias (28.1%, *n* = 286)**
Trial Fairness (55.4%, *n* = 563)	Race/Ethnicity (25.4%, *n* = 258)
Bias Education (41.1%; *n* = 418)	Gender/Sex (10.5%, *n* = 107)
Juror Duties (19.1%; *n* = 194)	Age (3.2%, *n* = 33)
Group-Based Disparities (10.5%, *n* = 107)	Socioeconomic Status (3.1%, *n* = 32)
Everyone is Biased (12.6%, *n* = 128)	Physical Appearance (2.8%, *n* = 28)
Verdict Accuracy (3.3%, *n* = 34)	Religious Identity (2.4%, *n* = 24)
Trust in Courts (3.1%; *n* = 31)	LGBTQ+ (2.3%, *n* = 23)
Jury Selection (2.2%; *n* = 22)	**Mentions of Race (25.3%, *n* = 257)**
Satisfying Litigants (2.0%; *n* = 20)	Racial Bias in Courts (11.7%, *n* = 119)
**Concerns and Criticisms (16.2%; *n* = 165)**	Systemic Racism (5.9%, *n* = 60)
Questioning Effectiveness (8.8%, *n* = 89)	Critical Focus on Race (1.3%, *n* = 13)
Unintended Consequences (3.2%, *n* = 33)	
Defensive Response (2.5%, *n* = 25)	

*Note.* This table reports the most frequent themes (bolded) for the full sample (*N* = 1016). Some infrequent subthemes are omitted here but discussed in the Supplemental Materials (pp. 16–18).

**Table A2:** Evaluation of specific intervention themes and subthemes.

**Content Takeaway (54.0%; *n* = 349)**
Conceptual Understanding of Implicit Bias (30.3%; *n* = 196)
Importance of Bias Awareness or Reflection (18.6%, *n* = 120)
Duties of a Juror (25.9%, *n* = 167)
Systemic Efforts of the Justice System Towards Fairness (2.5%, *n* = 16)
**Educational Value (45.8%; *n* = 296)**
New Concepts (30.0%; *n* = 194)
Prior Knowledge but Appreciated Reinforcement for **Self** (7.3%; *n* = 47)
Prior Knowledge but Appreciated Reinforcement for **Others** (1.4%; *n* = 9)
Concerns or Critiques about the Educational Value (8.8%; *n* = 57)
**Personal Reflection (6.7%; *n* = 43)**
Personal Bias Awareness (4.6%; *n* = 30)
Personal Change or Action Regarding Bias (2.8%; *n* = 18)
**Video Features—Overall (*Positive:* 52.5%, *n* = 339; *Negative:* 6.0%, *n* = 39; *Mixed:* 14.4%, *n* = 93)**
Delivery (*Positive:* 18.0%, *n* = 116; *Negative:* 6.3%, *n* = 41; *Mixed:* 0.9%, *n* = 6)
Clarity of Content (*Positive:* 14.6%, *n* = 94; *Negative:* 0.9%, *n* = 6; *Mixed:* 0.5%, *n* = 3)
Content (*Positive:* 17.5%, *n* = 113; *Negative:* 5.1%, *n* = 33; *Mixed:* 2%, *n* = 13)
Length/Pacing (*Positive:* 3.3%, *n* = 21; *Negative:* 2.0%, *n* = 13; *Mixed:* 0.6%, *n* = 4)
Examples (*Positive:* 7.3%, *n* = 47; *Negative:* 1.4%, *n* = 9; *Mixed:* 0.6%, *n* = 4)
Speakers (*Positive:* 3.6%, *n* = 23; *Negative:* 0.8%, *n* = 5; *Mixed:* 0.3%, *n* = 2)
Strategies (*Positive:* 10.7%, *n* = 69; *Negative:* 1.7%, *n* = 11; *Mixed:* 0.2%, *n* = 1)
Tone (*Positive:* 6.7%, *n* = 43; *Negative:* 1.9%, *n* = 12; *Mixed:* 0%, *n* = 0)

*Note.* The denominator of each major theme is *N* = 646, consisting of participants randomly assigned to watch an implicit bias intervention (educational video, *n* = 318; judicial instructions, *n* = 328)—and provided an open-ended response in which they evaluated the intervention.

**Table A3:** General perceptions of implicit bias interventions, split by political orientation.

	Total Sample	Liberals	Conservatives			
Item	*M* (*SD*)	*M* (*SD*)	*M* (*SD*)	*t*	*p*	*d*
Implicit bias interventions raise jurors’ awareness of their biases (+)	5.99 (1.18)	6.22 (0.83)	5.72 (1.43)	6.85	<0.001 **	0.43
Implicit bias interventions make trials more fair (+)	5.66 (1.35)	5.90 (0.96)	5.40 (1.65)	6.06	<0.001 **	0.38
Implicit bias interventions show people that the courts care about impartiality (+)	5.65 (1.29)	5.84 (1.02)	5.44 (1.51)	4.91	<0.001 **	0.31
Implicit bias interventions are necessary to prevent jurors’ biases from affecting their judgments (+)	5.61 (1.44)	5.90 (1.03)	5.29 (1.73)	6.91	<0.001 **	0.43
Implicit bias interventions are a way to make people or the courts feel better about themselves (−)	3.40 (1.78)	3.16 (1.66)	3.66 (1.87)	−4.45	<0.001 **	−0.28
Implicit bias interventions are a product of a political agenda, rather than science (−)	2.57 (1.74)	1.90 (1.20)	3.31 (1.93)	−14.18	<0.001 **	−0.89
Implicit bias interventions exaggerate a problem that, at best, minimally impacts jurors’ decisions (−)	2.52 (1.60)	2.00 (1.19)	3.09 (1.79)	−11.58	<0.001 **	−0.73
Implicit bias interventions are a waste of the court’s time and our tax dollars (−)	2.20 (1.49)	1.75 (1.00)	2.70 (1.76)	−10.80	<0.001 **	−0.68

** *p* < 0.01. *Note.* The valence of each item (positive or negative) is noted in parentheses for reference but was not shown to participants. Items, captured on a seven-point scale, are ordered by agreement across the sample. “Liberals” include participants who identified as slightly, somewhat, or very liberal, and “conservatives” include participants who identified as slightly, somewhat, or very conservative.

**Table A4:** Evaluations of educational videos and judicial instructions.

Outcome	Educational Video	Judicial Instructions	*t* (648)	*p*	*d*
	*M* (*SD*)	*M* (*SD*)			
To what extent was the video *helpful*?	3.77 (1.12)	3.68 (1.13)	1.02	0.31	0.08
To what extent was the video *informative*?	3.99 (1.01)	3.81 (1.04)	2.18	0.03 **	0.17
To what extent was the video *engaging*?	3.35 (1.07)	2.76 (1.13)	6.78	<0.001 **	0.53
To what extent was the video *confusing*?	1.13 (0.40)	1.14 (0.42)	−0.16	0.88	−0.01
To what extent was the video *unscientific*?	1.64 (0.98)	1.64 (0.93)	0.06	0.95	0.00
To what extent was the video *misguided*?	1.18 (0.60)	1.15 (0.56)	0.72	0.47	0.06

** *p* < 0.01.

**Table A5:** Pearson’s correlation matrix: support for implicit bias interventions and individual difference measures.

	1	2	3	4	5	6	7	8	9	10	11
Support for Implicit Bias Interventions (1)	—										
	—										
Psychological Reactance Scale (2)	−0.72	—									
	<0.001 **	—									
Deny Scale (3)	−0.42	0.43	—								
	<0.001 **	<0.001 **	—								
Defend Scale (4)	−0.40	0.44	0.62	—							
	<0.001 **	<0.001 **	<0.001 **	—							
Distance (Identity) Scale (5)	−0.06	0.08	0.34	0.12	—						
	0.04 *	0.01 *	<0.001 **	<0.001 **	—						
Distance (Inequality) Scale (6)	−0.27	0.32	0.67	0.47	0.38	—					
	<0.001 **	<0.001 **	<0.001**	<0.001 **	<0.001 **	—					
Dismantle Scale (7)	0.41	−0.38	−0.74	−0.50	−0.26	−0.53	—				
	<0.001 **	<0.001 **	<0.001 **	<0.001 **	<0.001 **	<0.001 **	—				
Implicit Racial Bias (8)	−0.02	0.04	0.14	0.13	0.00	0.12	−0.12	—			
	0.45	0.22	<0.001 **	<0.001 **	1.00	<0.001 **	<0.001 **	—			
Explicit Racial Bias (9)	−0.43	0.48	0.57	0.66	0.10	0.40	−0.55	0.11	—		
	<0.001 **	<0.001 **	<0.001 **	<0.001 **	0.002 **	<0.001 **	<0.001 **	<0.001 **	—		
Skepticism of Social Scientists Studying Race (10)	−0.55	0.55	0.55	0.44	0.17	0.38	−0.57	0.05	0.48	—	
	<0.001 **	<0.001 **	<0.001 **	<0.001 **	<0.001**	<0.001 **	<0.001 **	0.13	<0.001 **	—	
Political Conservatism (11)	−0.36	0.32	0.73	0.55	0.23	0.53	−0.65	0.13	0.47	−0.49	—
	<0.001 **	<0.001 **	<0.001 **	<0.001 **	<0.001 **	<0.001 **	<0.001 **	<0.001 **	<0.001 **	<0.001 **	—

** *p* < 0.01. * *p* < 0.05. *Note*. The top value in each row represents Pearson’s *r*, and the bottom value represents the associated *p* value.

**Table A6:** Individual difference measures predicting support for implicit bias interventions.

Predictor	*B*	*SE*	95% CI		*p*
			Lower	Upper	
Political conservatism	−0.04	0.02	−0.09	−0.001	0.04 *
Psychological reactance	−0.72	0.03	−0.78	−0.66	<0.001 **
Skepticism toward social scientists studying race	−0.19	0.03	−0.25	−0.13	<0.001 **
Defend strategy	−0.01	0.02	−0.06	0.03	0.52
Deny strategy	−0.01	0.03	−0.06	0.04	0.75
Distance inequality strategy	0.04	0.02	0.001	0.09	0.051
Distance identity strategy	0.03	0.02	−0.02	0.07	0.20
Dismantle strategy	0.05	0.02	0.001	0.09	0.04 *

** *p* < 0.01. * *p* < 0.05.
