# Supplementary material for: White Participants’ Perceptions of Implicit Bias Interventions in U.S. Courts
Source: Behav Sci (Basel). 2025 Sep 17;15(9):1269. doi: 10.3390/bs15091269 (PMC12466720; doi:10.3390/bs15091269)
Supplement: Supplementary file 1 [file behavsci-15-01269-s001.zip › behavsci-3696969-supplementary.pdf]

White Participants' Perceptions of Implicit Bias Interventions in U.S. Courts

Online Supplemental Materials

Author Note:

Stimulus materials, data, and analytic code are available on the Open Science Framework here.

Preregistration information is available here.

Table of Contents

Information About the Selected Interventions 3

Comparisons by Experimental Conditions 3

Plaintiff Race Condition 3

Implicit Bias Intervention Condition 8

Thematic Analysis of Open-Ended Responses 12

Thematic Analysis 1: General Perceptions of Implicit Bias Interventions 12

Thematic Analysis 2: Evaluations of Specific Implicit Bias Interventions 19

Supplemental Analyses: Quantitative Ratings 19

Information About the Selected Interventions

At the request of a reviewer, we provide two tables here that compare the content of the implicit bias interventions that we used in our experiment (educational videos: Table S1; judicial instructions: Table S2).

Comparisons by Experimental Conditions

The current article addresses a secondary question from a broader experiment that manipulated whether mock jurors (a) judged a case with a Black or White plaintiff and (b) watched an implicit bias intervention (educational video about implicit bias, judicial instructions about implicit bias, no intervention). As such, it is important to be mindful of whether these experimental conditions influenced the key measures relevant to the current paper, which focuses on perceptions of implicit bias interventions and individual differences that may be associated with those perceptions. We found little evidence that participants' judgments were influenced by the experimental manipulations.

Plaintiff Race Condition

We conducted t-tests to examine whether participants' responses to 24 total quantitative measures differed by their plaintiff race condition (Black, White). There were no significant differences across measures associated with participants' evaluations of the interventions (Table S3) nor measures capturing individual differences (Table S4) based on participants' plaintiff race condition.

Table S1.

Content Comparison of the Implicit Bias Educational Videos (Stimulus-Sampled)

Educational Video	Length (MM:SS)	Emphasis	Speakers	Tone	Distinct Content Features
Harris County District Clerk (2019)	06:47	Responsibility, awareness, disclosure	Judge, DA, defense attorney, district clerk	Narrative, personal, legal-professional	- Strong emphasis on bias disclosure during jury selection<br>- Increased focus on racial bias<br>- Includes personal stories and community-based examples
New Jersey Courts (2022)	06:48	Equal treatment, fairness, neutrality	Animated with voice-over narration	Formal, civic-duty tone	- Uses neutral, low-stakes examples (e.g., sports preferences)<br>- Focus on constitutional duty and fairness
New York State Unified Court System (2021)	10:38	Mental shortcuts and impact on fairness	Law professor, justice advocate (Perception Institute)	Educational, research-driven, inclusive	- Uses visual illusions and psychology-based demonstrations<br>- Highlights stereotype formation through media<br>- Increased focus on racial bias and identity
Western District of Washington (2021)	10:53	Conscious correction of unconscious bias	U.S. District Judge, attorneys	Reflective, practical, interactive	- Demonstrates scientific studies (e.g., Stroop task)<br>- Frames bias as automatic default to be overridden<br>- Emphasizes that good intentions are insufficient

Note. This table includes a combination of author-generated information (e.g., video length, identified speakers, and select distinct content features) and supplementary analysis using ChatGPT (OpenAI, 2023). We extracted full transcripts of each video using YouTubeToTranscript.com and input these transcripts to ChatGPT to offer an impartial content comparison of video elements that are more subjective (e.g., tone, emphasis). Of course, this is not a comprehensive list of the interventions' features. Each video defines and distinguishes between implicit and explicit bias, describes social and cognitive psychological findings, encourages jurors to self-reflect on their own bias, and emphasizes jurors' duty in their role.

Table S2.

Content Comparison of the Judicial Instructions (Stimulus-Sampled)

Judicial Instructions	Video Length (MM:SS)	Word Count	Tone	Distinct Content Features
American Bar Association (2017)	01:24	227	Measured, instructive, focused on mental control	- Uses metaphor of knee reflexes to explain automatic responses<br>- Encourages deliberate, conscious correction and counterfactual thinking
Minnesota v. Chauvin (2021)	02:24	345	Direct, morally urgent, socially aware	- Uses example of gender-career biases<br>- Discusses biases that favor those who are similar to us<br>- Provides expansive list of protected social categories (race, religion, SES, etc.)
New Jersey Courts (n.d.)	01:35	543	Formal, civic-minded, directive	- Legal framing of fairness as juror obligation<br>- Focus on weighing evidence calmly and without passion
New York State Unified Court System (2021)	01:55	323	Inclusive, educational, conversational	- Provides expansive list of protected social categories (race, religion, disability, etc.)<br>- Emphasizes the moral obligation to make decisions without bias

Note. As described in Table S1, this table includes a combination of author-generated information (e.g., video length, identified speakers, and select distinct content features) and supplementary analysis using ChatGPT (OpenAI, 2023).

Table S3.

Perceptions of Implicit Bias Interventions by Plaintiff Race Condition

Variable	Black Plaintiff		White Plaintiff					
	M	*SD*	*M*	*SD*	*t* statistic	*df*	*p*	Cohen's *d*
Support for implicit bias interventions	4.98	1.15	4.98	1.18	-0.03	1013.30	.98	0.00
Implicit bias interventions make trials more fair (+)	5.62	1.39	5.70	1.32	-0.94	1011.30	.35	0.06
Implicit bias interventions raise jurors' awareness of their biases (+)	5.94	1.22	6.03	1.13	-1.25	1007.20	.21	0.08
Implicit bias interventions show people that the courts care about impartiality (+)	5.68	1.24	5.62	1.34	0.81	1009.50	.42	0.05
Implicit bias interventions are necessary to prevent jurors' biases from affecting their judgments (+)	5.56	1.46	5.66	1.42	-1.06	1013.30	.29	0.07
Implicit bias interventions are a product of a political agenda, rather than science (-)	2.61	1.75	2.53	1.72	0.67	1013.50	.50	0.04
Implicit bias interventions are a way to make people or the courts feel better about themselves (-)	3.41	1.77	3.38	1.79	0.31	1013.90	.75	0.02
Implicit bias interventions are a waste of the court's time and our tax dollars (-)	2.22	1.51	2.17	1.47	0.51	1012.70	.61	0.03
Implicit bias interventions exaggerate a problem that, at best, minimally impacts jurors' decisions (-)	2.55	1.63	2.49	1.57	0.61	1012.20	.54	0.04
To what extent was the video informative?	3.90	1.02	3.90	1.04	-0.10	641.95	.92	0.01
To what extent was the video engaging?	3.01	1.13	3.10	1.15	-0.90	641.51	.37	0.07
To what extent was the video helpful?	3.71	1.14	3.75	1.11	-0.48	645.86	.63	0.04
To what extent was the video confusing?	1.11	0.37	1.16	0.44	-1.35	608.83	.18	0.11
To what extent was the video misguided?	1.15	0.55	1.18	0.61	-0.46	627.28	.65	0.04
To what extent was the video unscientific?	1.60	0.93	1.68	0.98	-1.10	637.00	.27	0.09

Note. A Welch's ANOVA was used to correct for unequal variances.

Table S4.

Individual Difference Measures by Plaintiff Race Condition

	Black Plaintiff		White Plaintiff					
Variable	*M*	*SD*	*M*	*SD*	*t* statistic	*df*	*p*	Cohen's *d*
Psychological Reactance Scale	2.32	0.95	2.33	0.94	-0.14	1013.77	.89	0.01
Skepticism of Social Scientists Scale	1.40	1.11	1.41	1.03	-0.08	1007.12	.94	0.00
Defend Scale	2.26	1.41	2.27	1.33	-0.11	1010.34	.91	0.01
Deny Scale	3.28	1.90	3.28	1.85	0.04	1013.09	.97	0.00
Distance Inequality Scale	3.80	1.52	3.77	1.50	0.28	1013.60	.78	0.02
Distance Identity Scale	5.15	1.27	5.1	1.25	0.64	1013.57	.52	0.04
Dismantle Scale	3.90	1.74	3.86	1.76	0.35	1013.97	.72	0.02
Explicit Racial Bias	2.94	1.30	2.94	1.23	0.03	1008.66	.97	0.00
Implicit Racial Bias	0.44	0.40	0.45	0.37	-0.16	996.32	.87	0.01
Psychological Reactance Scale	2.32	0.95	2.33	0.94	-0.14	1013.77	.89	0.01

Note. A Welch's ANOVA was used to correct for unequal variances.

Implicit Bias Intervention Condition

We also examined whether participants' responses varied by their implicit bias intervention condition. As noted in the article, participants who viewed either intervention (judicial instructions, educational video) expressed greater support for their use in courts than participants who did not view an intervention. This pattern may reflect that participants unfamiliar with these interventions feel less confident about supporting their use in courts or draw from their prior experiences or attitudes toward diversity, equity, and inclusion trainings, which can be quite polarizing (Minkin, 2023; Palmer, 2024; Sheen, 2023). In addition, we compared participants' evaluative ratings about the specific intervention they watched (if applicable) across six measures (see Table A4 in the article).

We also conducted between-subjects ANOVAs to examine whether participants' responses on the 17 remaining quantitative measures assessing general impressions of implicit bias interventions (Table S5) and individual differences (Table S6) differed by the implicit bias intervention condition. Only one individual difference measure differed by intervention condition: participants' self-reported psychological reactance. Participants who did not watch an intervention reported greater psychological reactance (M = 2.44, SD = 0.97) than participants who watched an educational video (M = 2.22, SD = 0.92). There were no significant differences in reported psychological reactance for participants in the judicial instructions about implicit bias condition (M = 2.30, SD = 0.91) relative to those who watched an educational video about implicit bias, p = .54, or who did not receive an implicit bias intervention, p = .11.

The finding that participants in the educational video condition reported less psychological reactance (vs. no intervention) is to be expected. This scale consisted of three modified measures from previous research (Lofaro et al., 2024) and three novel, face-valid items to capture participants' perceptions of whether implicit bias interventions are useful for them (e.g., "Although some might need these kinds of implicit bias interventions, I don't"). The educational videos emphasize that everyone is subject to implicit biases, which may have reduced resistance to bias education. Although judicial instructions about implicit bias have similar themes, they spend less time on these topics, which might explain why this effect did not extend to participants in the judicial instructions condition.

Table S5.

Perceptions of Implicit Bias Interventions by Implicit Bias Intervention Condition

	Educational Video		Judicial Instructions		No Intervention				
Variable	*M*	*SD*	*M*	*SD*	*M*	*SD*	*F* statistic	*df*	*p*
Support for implicit bias interventions	5.09	1.14	5.08	1.11	4.78	1.21	7.77	2, 672.87	< .001*
Implicit bias interventions make trials more fair (+)	5.70	1.32	5.71	1.31	5.59	1.42	0.84	2, 673.16	.43
Implicit bias interventions raise jurors' awareness of their biases (+)	6.07	1.12	6.04	1.18	5.86	1.22	3.01	2, 672.95	.05
Implicit bias interventions show people that the courts care about impartiality (+)	5.72	1.20	5.72	1.22	5.53	1.42	2.43	2, 675.04	.09
Implicit bias interventions are necessary to prevent jurors' biases from affecting their judgments (+)	5.67	1.39	5.67	1.39	5.50	1.53	1.58	2, 673.86	.21
Implicit bias interventions are a product of a political agenda, rather than science (-)	2.45	1.70	2.50	1.73	2.74	1.77	2.69	2, 671.75	.07
Implicit bias interventions are a way to make people or the courts feel better about themselves (-)	3.40	1.85	3.29	1.71	3.49	1.78	1.18	2, 668.69	.31
Implicit bias interventions are a waste of the court's time and our tax dollars (-)	2.16	1.46	2.10	1.44	2.33	1.55	2.19	2, 672.89	.11
Implicit bias interventions exaggerate a problem that, at best, minimally impacts jurors' decisions (-)	2.43	1.55	2.48	1.59	2.62	1.65	1.40	2, 672.43	.25

Note. A Welch's ANOVA was used to correct for unequal variances.

*$p < .001$

Table S6.

Individual Difference Measures by Implicit Bias Intervention Condition

Variable	Educational Video		Judicial Instructions		No Intervention					
	M	*SD*	*M*	*SD*	*M*	*SD*	*F* statistic	between *df*	within *df*	*p*
Psychological Reactance Scale	2.22	0.92	2.30	0.91	2.44	0.97	4.76	2	672.59	.009*
Skepticism of Social Scientists Scale	1.30	1.06	1.45	1.08	1.46	1.07	2.33	2	669.64	.10
Defend Scale	2.22	1.36	2.27	1.35	2.30	1.40	0.32	2	671.67	.72
Deny Scale	3.24	1.87	3.32	1.85	3.27	1.90	0.16	2	670.83	.85
Distance Inequality Scale	3.71	1.54	3.80	1.48	3.84	1.51	0.66	2	669.49	.52
Distance Identity Scale	5.08	1.22	5.20	1.25	5.09	1.30	0.93	2	672.54	.39
Dismantle Scale	3.99	1.75	3.82	1.69	3.83	1.79	0.99	2	671.24	.37
Explicit Racial Bias	2.90	1.26	2.91	1.25	3.00	1.29	0.66	2	670.54	.52
Implicit Racial Bias	0.44	0.39	0.43	0.37	0.46	0.39	0.40	2	664.42	.67

Note. A Welch's ANOVA was used to correct for unequal variances.

*$p < .01$

Thematic Analysis of Open-Ended Responses

Thematic Analysis 1: General Perceptions of Implicit Bias Interventions

As stated in the main text, we conducted a thematic content analysis to explore patterns in participants' responses to two open-ended questions, the first of which explored their general perceptions of implicit bias interventions ("What do you think motivated the courts to adopt these interventions? What are your opinions and thoughts about the use of implicit bias interventions in the courts?"). Here, we provide greater detail about the iterative coding process for this question, report agreement for the two independent coders, and dive deeper into participants' responses beyond what is reported in the article.

Iterative Coding Process

We used OpenAI (2023) to identify initial patterns in participants' responses. We used these suggestions as a foundation to develop the codebook, manually skimmed through responses, made adjustments to capture additional themes that were theoretically relevant to our team (e.g., verdict accuracy; the concept that we all hold biases; counterfactual thinking techniques), and ensured that each category was conceptually distinct. Following this initial codebook development process, two independent coders evaluated one participant response at a time before checking for agreement on each code. This ensured that as the coding process developed, coders were able to explain their use of certain codes when discrepancies arose, converge in the way that they were thinking about each code, and revise the codebook when necessary.

Because thematic content analyses largely intend to capture themes that are frequently occurring in the data (Braun & Clarke, 2006), after the coders systematically examined approximately 50 responses, the coders removed those that were particularly rare. For example,

coders initially recorded instances where mock jurors referenced various sources of their own biases (e.g., media, schooling, members of their community) or counterfactual thinking techniques (i.e., imagining that a litigant was of a different demographic group). These codes were removed due to their infrequency. Coders also added subthemes that emerged in these initial 50 responses, such as reference to juror biases against litigants based on their physical appearance (e.g., clothing; subtheme of *targets of bias*).

Coders also reassessed the codebook when lack of clarity between codes were causing significant and consistent discrepancies. For instance, the codebook included two broad codes that differentiated between participants' beliefs about (a) the court's motivation for adopting the interventions and (b) what jurors would take away from the interventions. In addition, the codebook initially distinguished between reasons *why* participants believed the interventions were of educational value—for example, whether they increased jurors' awareness of their own biases, encouraged jurors to take their role of being unbiased more seriously, or motivated jurors to slow down their decision-making. However, these themes overlapped substantially, making it challenging for coders to achieve acceptable reliability. After coding 150 participant responses, the coders discussed the issues with distinguishing between participants' perceptions of the court's motivations and their personal beliefs about the intervention's educational value. As a result, coders merged the various reasons why participants believed the interventions were of educational value into a single code reflecting *bias education* and decided to narratively describe common thoughts that participants expressed related to the interventions' educational value instead.

Throughout their progression through the dataset, coders occasionally noted the general occurrence of emerging themes, without capturing their precise frequency due to time

constraints. For example, coders noted explicit statements that not everyone is biased, as well as references to social science about bias and/or disparities. As a result, these are described narratively in the main article.

Finally, after coders completed all responses, they then made judgments about which codes to remove from the formal analyses due to their infrequency across the entire sample, including codes capturing personal experiences related to race/racial bias (within broader theme *mentions of race*) and resource concerns (i.e., concerns about the time, cost, or feasibility of implementing the interventions; within broader theme *concerns & criticisms*).

AI Generated Themes by Plaintiff Race Condition.

In response to a request by a reviewer, we also explored whether the AI-generated themes differed meaningfully between participants in each plaintiff race condition. We created two separate datasets: one containing responses from each plaintiff race condition (Black, White). For each dataset, we prompted ChatGPT to summarize each response, extract the top ten major themes and briefly describe each. In a new chat, we uploaded the new themes and descriptions, split by plaintiff race condition, and prompted ChatGPT to generate a comparison table highlighting similarities and differences between the themes in these two datasets (see Table S7). ChatGPT concluded that the datasets exhibited strong thematic convergence with some slight differences in tone or emphasis in the themes. To be clear, these themes were largely overlapping, but separate, from the full-sample AI-generated themes we summarize in the main text.

Table S7.

AI-Generated Themes by Plaintiff Race Condition (Thematic Analysis 1)

Themes for Black Plaintiff Condition	Themes for White Plaintiff Condition	Theme Comparison
Raising Awareness of Implicit Bias	Raising Juror Awareness of Implicit Bias	Same core idea
Preventing Stereotypical Judgments	Courts Addressing Bias Through Structured Interventions	Similar goal, different framing
Promoting Deliberate and Fair Trials	Promoting Fairness and Reducing Bias in Trials	Same intention, wording differs
Responding to Historical Injustices	Responding to Historical or Evidenced Injustices	Very similar
General Support for Interventions	General Support for Implicit Bias Interventions	Nearly identical
Improving Juror Decision-Making	Improving Decision-Making by Minimizing Bias	Very similar concept
Encouraging Personal Bias Recognition	Encouraging Personal Reflection and Bias Recognition	Same theme with varied wording
Addressing Demographic Biases	Confronting Racial and Demographic Bias in Courtrooms	Very similar content
Ensuring Fairness and Impartiality	Concern About Bias Undermining Justice	Same focus, different emphasis
Motivations for Court Adoption	Belief That Interventions Are Necessary and Helpful	Overlap in purpose, framed differently

Percent Agreement Between Coders by Theme

Two independent coders showed acceptable agreement across themes about general perceptions of implicit bias interventions in the first question (*M* = 96.1%; *median* = 98.7%; *range* = 75.5% – 99.8%; see Table S8).

Table S8.

Percent Agreement for Thematic Analysis 1

**Court Motivation**	**Targets of Bias**
Trial Fairness: 75.5%	Race/Ethnicity: 98.0%
Bias Education: 85.1%	Gender/Sex: 98.9%
Juror Duties: 86.1%	Age: 99.6%
Group-Based Disparities: 93.5%	Socioeconomic Status: 99.3%
Everyone is Biased: 98.8%	Physical Appearance: 99.0%
*Verdict Accuracy: 98.2%	LGBTQ+: 99.8%
*Trust in Courts: 98.2%	Religious Identity: 99.8%
*Jury Selection: 98.6%	**Mentions of Race**
*Satisfying Litigants: 99.2%	Racial Bias in Courts: 95.4%
**Concerns & Criticisms**	Systemic Racism: 95.8%
Questioning Effectiveness: 98.0%	Critical Focus on Race: 99.0%
Unintended Consequences: 99.1%	
Defensive Response: 98.7%	

Note. An asterisk (*) indicates that a subtheme is fully reported here, rather than in the article. Initial percent agreement between the two coders was calculated for each individual subtheme. For example, if coders initially disagreed about whether a participant referenced "Unintended Consequences," a subtheme under the broader theme Concerns and Criticisms, that discrepancy was recorded only for the "Unintended Consequences" subcode and did not affect agreement for other subcodes (e.g., "Questioning Effectiveness").

Description of Relatively Infrequent Themes

In the article, we fully describe the most common themes that emerged from our coding. For brevity, we simply referenced the less frequent subthemes that we captured—including *verdict accuracy*, *trust in courts, jury selection, satisfying litigants,* and a few others—which we fully describe here.

Some participants indicated that the interventions were intended to increase the accuracy of verdicts (3.3%, n = 34; i.e., *verdict accuracy*) by reducing wrongful convictions and/or preventing guilty individuals from going free. One participant, for example, attributed the courts' motivation to the prevalence of "innocent African Americans that are in prison and have later been exonerated." Others framed it as a product of growing media attention and public awareness, noting that the proliferation "of documentaries that have come out about wrong convictions and judgements in recent years are waking many people up to the fact that our court systems are far from perfect." Alternatively, some participants focused on verdict errors more broadly, suggesting that what "motivated the court is the unconscious and involuntary mistakes or errors made by jurors."

Moreover, several participants believed that the interventions were adopted to promote the public's trust and confidence in the courts (3.1%; n = 31; i.e., *trust in courts*). For example, one participant expressed the view that the court is "only doing its due diligence to maintain the court's legitimacy in the public eye" by adopting the interventions. Sometimes, this was framed as addressing public dissatisfaction with the courts; for example, one participant stated that the interventions are "in response to public scrutiny and demands for more equitable justice practices."

Others framed the intervention as a tool to assist in seating an impartial jury (2.2%; n = 22; i.e., *jury selection*). These participants often referenced the importance of "weed[ing] out" jurors who are biased and believed that jurors may be more willing to publicly share their biases when the information in the interventions is "brought to their attention." A few participants made note of the interventions' goal to normalize bias. For example, one participant noted that an implicit bias intervention gives the opportunity for jurors to share their biases "while also telling them…that they won't be judged for coming forward with their bias."

A handful of participants claimed that the interventions were adopted to prevent litigants from challenging a verdict that is unfavorable to them (2.0%; n = 20; i.e., *satisfying litigants*). These participants often noted that the interventions aimed to prevent appeals, mistrials, or lawsuits based on juror bias or assumed that they could be used in trial at the litigants' request. For example, one participant believed they were adopted because of "people claiming to [have] lost their cases because the jury was biased against them… It may also be an attempt to reduce the strain on the appellate system."

Finally, we captured "other" references to the courts' motivation that, though infrequent (less than 20 responses), nevertheless captured some themes that we felt were particularly relevant to the U.S. climate today. For example, participants sometimes referenced that emerging findings in social science, particularly, led to the adoption of implicit bias interventions (e.g., referencing mock juror studies that demonstrate bias by manipulating litigant characteristics, like race). Others referenced the general shift across sectors (e.g., companies, government entities, educational settings) toward discussing explicit and implicit biases. Some of these participants discussed the interventions in the context of DEI initiatives more broadly (e.g., relating to the frequency at which DEI initiatives are found in the workplace). Relatedly, some participants

argued that the interventions were necessitated by increasing polarization in the U.S. (e.g., "I think the adoption of these interventions was motivated by societies seemingly increasing division of opinions and biases"; "The divide over everything in society is probably why they have to read these instructions"). Finally, a handful of participants stated that peoples' biases have been exacerbated by social media (e.g., describing social media as "echo chambers that can begin or strengthen implicit bias toward certain groups of people").

Thematic Analysis 2: Evaluations of Specific Implicit Bias Interventions

As stated in the main text, we conducted a thematic content analysis to explore patterns in participants' responses to two open-ended questions, the second of which was only asked to participants who were randomly assigned to watch one of the interventions (i.e., judicial instructions or educational video) and explored participants' evaluations of the specific implicit bias intervention video they watched ("In a few sentences, what was your impression of this video? What did you learn or take away from the video?"). Here, we provide greater detail about the iterative coding process for this question, report agreement for the two independent coders, and dive deeper into participants' responses beyond what is reported in the manuscript.

Iterative Coding Process

As stated in the main text, we first used OpenAI's ChatGPT (2023) to assist in identifying and refining categories for the codebook. Specifically, we asked the AI to extract themes from manageable batches of participant responses. From these outputs, we selected themes that aligned with our team's research interests, including those related to participants' personal takeaways, general impressions, and perceived educational value of the interventions. We then posed follow-up questions to the AI to explore these themes in greater depth—for example, asking what participants liked (e.g., clear explanations, use of real-world examples),

what they disliked (e.g., dry delivery, repetitive content), and what key messages they took away (e.g., recognizing the widespread nature of bias). In addition, we manually skimmed through responses to identify emergent patterns, such as references to prior knowledge about implicit bias and distinctions between bias expression versus bias awareness and asked targeted questions to further refine these themes. We then created a codebook that included each of these themes and subthemes, the codes associated with each, and examples for reference while coding.

Following this initial codebook development process, two independent coders evaluated one participant response at a time before checking for agreement on each code. This ensured that as the coding process developed, coders were able to converge in the way that they were thinking about each code and could justify their use of certain codes when discrepancies arose. After coding approximately 50 participant responses, the coders needed to reassess the codebook due to a lack of clarity between codes causing significant and consistent discrepancies. Specifically, the coders failed to converge when some codes were conceptually overlapping (e.g., “Conceptual Understanding of Implicit Bias” vs. “Bias as Widespread/Pervasive”) or generally unclear (e.g., what constated as educational value).

This led to an in-depth discussion between the two coders about these issues and resulted in the coders together reevaluating the codebook to make certain codes clearer and avoid conceptual overlap (e.g., combining “Conceptual Understanding of Implicit Bias” and “Understanding of Bias as Widespread/Pervasive” under Content Takeaway) and establish objective standards for codes (e.g., words like “learn,” “taught,” “take away,” and “helped/made understand” all signify new knowledge and should be coded as such under Educational Value). The coders also added the code category “Content” into the Video Features section to help

differentiate between participants' responses that mentioned the quality, delivery, and clarity of the video content.

With these more clearly defined codes, the independent coders began the coding process again, including coding each individual participant's response before checking for consistency between codes, discussing any discrepancies between coders, and deciding on a final code. Once the coders were comfortable with these coding procedures and minimized discrepancies between codes, coders increased to three lines between discussion and then five lines between discussion. Finally, after coding chunks of five lines with minimal discrepancies, the coders began coding in groups of ten lines until they finished coding all participant responses.

Throughout the process of coding, the coders refined the codebook based on new issues, phrasing, or ideas that appeared in participant responses. For instance, after one participant mentioned that the videos were "well done," the coders jointly decided that this would be a positive code in the "Delivery" category (relative to "Content," "Clarity," or "Overall Video Features") and thus any subsequent mentions of the video being "well done" would fall under "Delivery." This allowed the coders to continue to solidify their understanding of each individual coding category while also adapting to include participant responses. Also of note, at approximately the halfway point of coding, the coders made a decision on the category "Strategies" to make it more exclusive (i.e., the participant needed to make it clear that they learned the strategy from the video). This required the coders to go back through each already coded response and reassess if the current codes that indicated mentions of strategies met this new criterion. The coders carried this standard throughout the rest of the participant responses.

AI Generated Themes by Plaintiff Race Condition.

In response to a request by a reviewer, we again explored whether the initial themes generated by AI differed meaningfully between participants in each plaintiff race condition. We uploaded separate datasets for each condition (Black, White) into ChatGPT, which summarized each response, extracted the top ten major themes, briefly described each, and generated a comparison table highlighting similarities and differences between the themes in these two datasets (see Table S9). ChatGPT again concluded that the datasets exhibited strong thematic convergence with some slight differences in tone or emphasis in the themes.

Table S9.

AI-Generated Themes by Plaintiff Race Condition (Thematic Analysis 2)

Theme Area	Black Q2 Theme Label	White Q2 Theme Label	Similarity/Difference
Bias Awareness	Bias Awareness	Focus on Self-Awareness and Personal Reflection	Similar – Both highlight increased awareness of personal bias prompted by the intervention.
Educational Purpose	Educational Value	Educational Value of Interventions	Similar – Both note the informative role of the intervention in teaching about bias.
Video Presentation	Video Quality and Presentation	Mixed or Neutral Reactions to Video Delivery	Similar – Both reference video clarity, tone, and presentation; tone of response differs.
Credibility of Messenger	Judicial Authority and Credibility	Praise for Judicial System Modernization	Partially Similar – Black Q2 focuses on the judge's credibility; White Q2 on institutional evolution.
Mandated Tone	Procedural Formality or Obligation	Concerns About Politicization or Overreach	Partially Similar – Both show skepticism, but White Q2 highlights political concerns.
Relevance to Jury Role	Application to Jury Duty	Support for Fairness and Equity	Partially Similar – Black Q2 focuses on juror behavior; White Q2 emphasizes justice outcomes.
Doubt or Dismissal	Skepticism or Resistance	Skepticism or Resistance Toward Effectiveness	Similar – Both express doubt about the intervention's impact.
Social Context	Social Issues and Stereotyping	Empathy and Concern for Marginalized Groups	Similar – Both reflect on broader societal biases and the need to protect vulnerable groups.
Call to Action	Call for Greater Change	Calls for Broader Systemic Change	Similar – Both call for deeper reforms beyond the current intervention.
Introspection	Personal Reflection	Recognition of Implicit Bias in Justice System	Partially Similar – Black Q2 emphasizes personal introspection; White Q2 adds institutional critique.

Percent Agreement Between Coders by Theme

As stated in the main text, two independent coders showed acceptable agreement across themes about perceptions of specific implicit bias interventions in the second question (*M* = 92.9%; *median* = 95.1%; *range*: 76.9% – 98.6%). Here, we report agreement for each specific theme (Table S10).

Table S10.

Percent Agreement for Thematic Analysis 2

Theme	Agreement
Content Takeaway	76.9%
Educational Value	88.0%
Personal Reflection	96.8%
Video Features – Overall	88.3%
Delivery	94.8%
Clarity of Content	95.1%
Content	87.8%
Length / Pacing	98.6%
Examples	98.5%
Speakers	98.3%
Strategies	89.8%
Tone	97.4%

Note. Initial percent agreement between the two coders was calculated based on agreement across all subthemes within each overarching theme. For example, the theme *Content Takeaway* included four subcodes: "conceptual understanding of implicit bias," "importance of bias awareness or reflection," "duties of a juror," and "systemic efforts of the justice system towards fairness." If the coders disagreed on any of these subcodes, the entire theme was counted as discrepant—even if they agreed on the other subthemes. Coders did not calculate reliability at the level of individual subthemes. As a result, the reported percent agreement likely underestimates the coders' actual level of reliability.
